# Reverse Micelles: The Gateway for Oral (Poly)Peptide Delivery

**DOI:** 10.1002/smll.76409

**Published:** 2026-10-24

**Authors:** Marlene Ramona Schmidt, Melanie Lena Ebert, Andreas Bernkop‐Schnürch

**Affiliations:** ^1^ Department of Pharmaceutical Technology Center For Chemistry and Biomedicine Institute of Pharmacy University of Innsbruck Innsbruck Austria

**Keywords:** dry reverse micelles, hydration state, lipid‐based formulations, oral peptide delivery, reverse micelles, surfactant packing parameter

## Abstract

Reverse micelles (RM) have played a central role in biotechnology, yet their pharmaceutical use has been constrained by instability, high water content and non‐biocompatible surfactants. Classical wet RM (wRM) contain a discrete aqueous core whose size and solubilization behavior are governed by surfactant packing, hydration level and physicochemical properties of the hydrophobic phase. Although these systems provided insights into nanoconfined environments, their sensitivity to dilution and environment has limited translation into drug delivery. Recent developments renew interest in dry RM (dRM), which reduce internal water to below 1% and rely mainly on interfacial rather than bulk‐like water domains, with electrostatic and non‐covalent interactions contributing to stabilization of highly hydrophilic (poly)peptides (pP). dRMs expand the application space for pP therapeutics, supporting delivery routes requiring low hydration and robust microenvironments. This review integrates RM architecture and outlines performance criteria for oral pP delivery, including high loading capacity, increased pP lipophilicity, protection from enzymatic degradation, enhanced epithelial permeation and favorable safety. In vivo studies demonstrate that dRM achieve comparable or improved oral bioavailability relative to hydrophobic ion pairs or wRM in selected systems, underscoring why RM remain relevant and why maintaining minimal hydration and formulation flexibility are central to unlocking their therapeutic potential.

## Graphical abstract

RM achieve superior oral peptide delivery through high loading capacity, strong lipophilicity, and a low‐hydration microenvironment that protects against enzymatic degradation. Meanwhile, dRM provides favorable permeation‐enhancing interactions and a consistent safety profile, collectively driving enhanced oral performance.

**Abbreviations**
*N_s_*
: Packing parameter
AOT: Sodium bis(2‐ethylhexyl) sulfosuccinate
API: Active pharmaceutical ingredients
ARM: Anhydrous reverse micelles
BA: Bioavailability
BHDC: Benzyldimethylhexadecylammonium chloride
bmimBF_4_
: 1‐butyl‐3‐methylimidazolium tetrafluoroborate
bmimTf2N: 1‐butyl‐3‐methylimidazolium bis(trifluoromethylsulfonyl)imide
Brij 93: Polyethylene glycololeylether
BSA: Bovine serum albumin
Capmul MCM C8 EP/ NF: Mono‐ and diglycerides of caprylic acid
Capmul PG‐2L: Propylene glycol dilaurate
c_max_
: Maximum plasma concentration
CMC: Critical micelle concentration
DA: Dry addition
DDAB: Didecyl dimethyl ammonium bromide
DMA: *N,N*‐Dimethylacetamide
DMF: *N,N*‐Dimethylformamide
DMSO: Dimethyl sulfoxide
DODAB: Dimethyl‐dioctadecyl‐ammonium bromide
DOTAP: 1,2‐dioleoyl‐3‐trimethylammonium‐propane
dRM: Dry reverse micelles
EE: Encapsulation efficiency
EG: Ethylene glycol
ELA: Ethyl lauroyl arginate
Exe: Exenatide
F_a_
: Absolute bioavailability
FL: Fluorescein
F_r_
: Relative bioavailability
GMCC: Glycerol monocaprylocaprate
GML: Glycerol monolinoleate
GRAS: Generally Recognized As Safe
GY: Glycerol
HA: Hyaluronic acid
HIP: Hydrophobic ion pairing
HRP: Horseradish peroxidase
IL: Ionic liquids
IPM: Isopropyl myristate
Labrafac MC60: Glycerol monocaprylocaprate
Labrafac Lipophile WL 1349: Cwaprylic/capric triglycerides
LNPs: Lipid‐based nanoparticles
Maisine CC: Glycerol monolinoleate
MCT: Medium‐chain triglycerides
MD: Molecular dynamics simulations
Na: Sodium
NLC: Nanostructured lipid carriers
NMR: Nuclear magnetic resonance spectroscopy
O/W: Oil‐in‐water
OS: Organic solvent
PEG: Polyethylene glycol
PG: Propylene glycol
PK: Pharmacokinetic
PMB: Polymyxin B
pP: (Poly)peptides
QAs: Quality attributes
RH40: Polyoxyl 40 hydrogenated castor oil (Kolliphor RH40)
RM: Reverse micelles
SANS: Small‐Angle Neutron Scattering
SAXS: Small‐Angle X‐ray Scattering
SEDDS: Self‐emulsifying drug delivery systems
SL: Soy lecithin
SLN: Solid lipid nanoparticles
Span 80: Sorbitan monooleate
t_max_
: Time to maximum plasma concentration
TOMAC: Trioctylmethylammonium chloride
Tween 85: Polyoxyethylen‐sorbitan‐trioleat
TX‐100: Triton X‐100
W/O: Water‐in‐oil
w₀: Water‐to‐surfactant ratio
wRM: Wet reverse micelles

## 1 Introduction

The concept of reverse micelles (RM) has its origins in mid‐20th‐century studies on water‐in‐oil emulsions. Early work by Schulman and co‐workers in the 1950s first demonstrated that surfactants in nonpolar media can organize into nanoscale structures capable of hosting confined water pools [1]. Subsequent physicochemical investigations by Scriven, Davis, Nagarajan and Ruckenstein (1960s–1970s) established RM as discrete supramolecular assemblies governed by interfacial packing and limited hydration [2, 3, 4]. A major conceptual consolidation occurred in the 1970s–1980s through the work of Paolo L. Luisi, who introduced RM as controllable nanoconfined aqueous environments and demonstrated their utility as model systems for enzymatic catalysis, solubilization phenomena and interfacial organization [5].

Building on these early conceptual foundations, modern RM are understood as nanoscale assemblies of amphiphilic surfactants in nonpolar media that create a confined polar interior capable of solubilizing water and hydrophilic molecules. Their internal organization is governed by hydration level, typically expressed as the molar water‐to‐surfactant ratio (w_0_), which dictates the balance between interfacial and bulk‐like water states and thereby controls solute mobility, reaction kinetics and biomolecular stability [6, 7, 8, 9]. RM differ from water‐in‐oil (w/o) microemulsions not in an absolute structural sense, but through an operational definition governed primarily by the molar water‐to‐surfactant ratio (w_0_ = [H_2_O] /[Surfactant]) and the resulting physicochemical state of the encapsulated water. Building on the classical descriptions by Luisi and Straub as well as Maitra, RM represent molecularly discrete, curvature‐dominated assemblies constrained by low hydration (w_0_ ≲ 10−15). In this regime, water molecules are strictly nanoconfined and predominantly bound to the surfactant headgroups, lacking true bulk‐like properties [10, 11].

In contrast, w/o microemulsions represent thermodynamically stable swollen phases—strictly distinct from kinetically stable nanoemulsions—occurring at higher *w*_0_ values, accommodating larger aqueous cores characterized by a distinct central region of free, bulk‐like water [10, 12, 13]. The high interfacial curvature and minimal hydration of RM thus position them as the limiting case at the high‐curvature end of the w/o structural spectrum—a concept explicitly framed by Ruckenstein and Nagarajan [14]. By defining the boundary operationally via w_0_ and the emergence of core bulk water, the structural continuity between wet RM and conventional w/o microemulsions is reconciled with their distinct local physicochemical environments.

Classical wet reverse micelles (wRM) contain a discrete aqueous core and exhibit rapid fusion–fission dynamics that facilitate equilibration but also render them structurally fragile. Their strong dependence on water content, temperature and dilution, together with the frequent use of non‐biocompatible surfactants, has historically limited their translation into pharmaceutical delivery systems [8, 15]. Under gastrointestinal conditions, wRM are prone to dilution‐induced disassembly or uncontrolled restructuring, compromising their ability to retain polar payloads.

Dry reverse micelles (dRM) represent a functional evolution of RM architecture. By reducing internal water to below an operational threshold of 1%, dRM—as observed in systems like Span‐based formulations—eliminate bulk‐like aqueous domains while retaining a polar interfacial microenvironment capable of stabilizing hydrophilic or charged species [16, 17, 18, 19]. Suppressed internal mobility and reduced fusion–fission dynamics confer markedly improved robustness under pharmaceutically relevant conditions, including resistance to dilution and compatibility with lipid‐based formulations. These features make dRM particularly attractive for the delivery of highly hydrophilic compounds—especially (poly)peptides (pP)—that remain challenging to incorporate into conventional lipid carriers. Acting as nanoscopic interfacial mediators, dRM enable the assembly of polar molecules with lipophilic matrices without chemical modification [17, 18, 19].

Despite growing interest in dRM, the field remains fragmented: fundamental studies on RM structure and dynamics often remain disconnected from applied formulation research and the transition from wRM to dRM has not been systematically conceptualized in terms of thermodynamics, interfacial organization or behavior under dynamic physiological conditions. A unified physicochemical framework is therefore needed to connect classical RM principles with emerging formulation strategies and translational requirements.

This review aims to provide such a framework by integrating fundamental concepts of RM and dRM organization with preparation as well as characterization strategies and by evaluating their performance within lipid‐based delivery platforms under biorelevant conditions. In doing so, it highlights the potential of dRM as next‐generation tools for the oral delivery of pP and other hydrophilic therapeutics.

## 2 Components of RM

RM consist of several interdependent structural elements whose collective organization defines curvature, stability and payload association. Figure 1 provides an overview of these key components and their spatial arrangement within the RM architecture.

**FIGURE 1:**
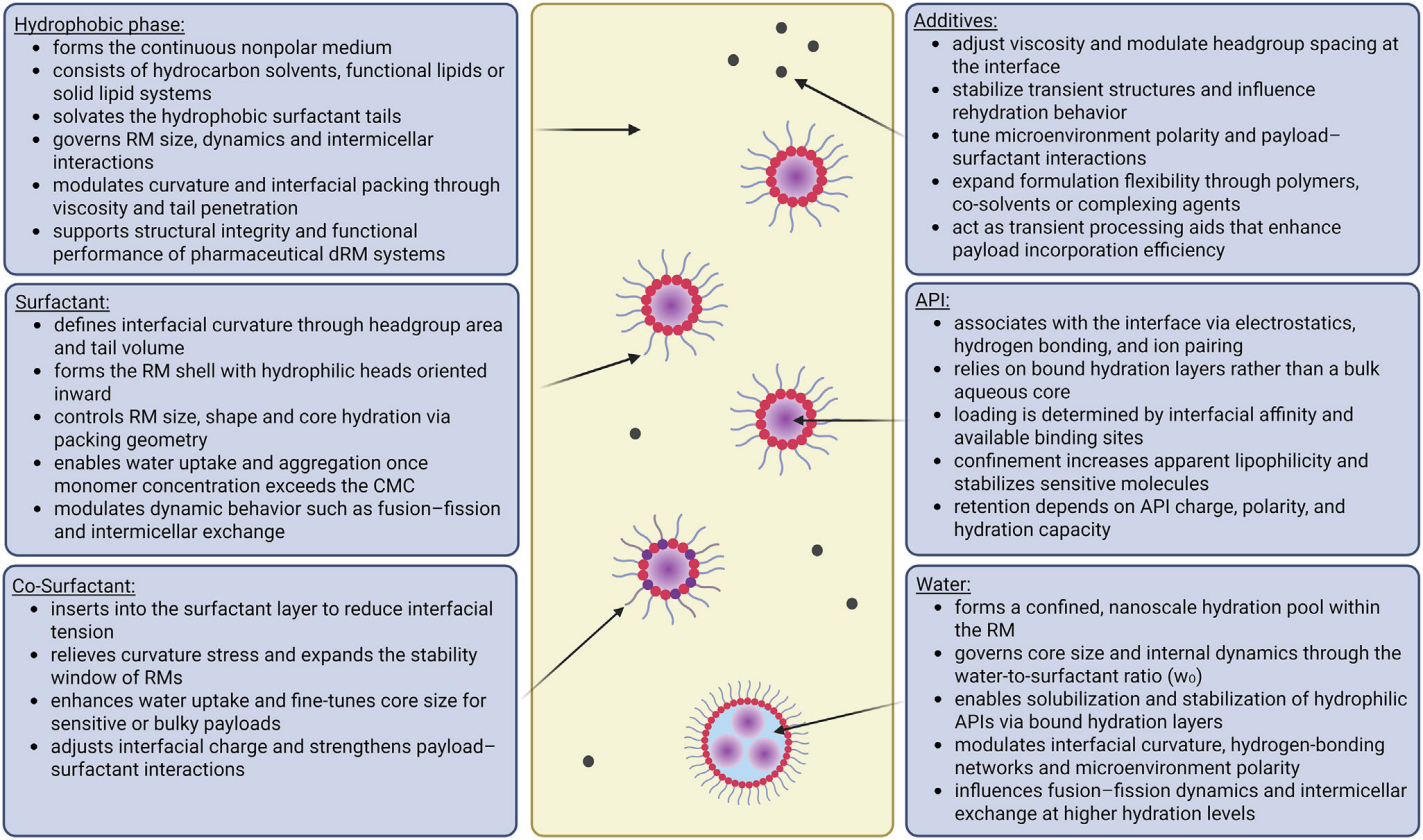
Overview of reverse micelle (RM) architecture, illustrating the roles and spatial organization of continuous hydrophobic phase, surfactant, co‐surfactant, water, additives and active pharmaceutical ingredients (API) within the RM structure.

### 2.1 Continuous Hydrophobic Phase

The nature of the hydrophobic phase plays a non‐negligible role in RM formation and stability [10]. Classical RM systems were typically formulated in simple hydrocarbon solvents such as isooctane or short‐chain alkanes, which enabled fundamental insights into RM structure but offered limited pharmaceutical relevance. Beyond these limitations, solvent properties such as dielectric constant, viscosity and molecular penetration into the surfactant tail region strongly influence RM size and intermicellar interactions [20]. Short‐chain alkanes generally promote smaller, more dynamic RM, whereas longer‐chain or more viscous lipid phases can stabilize larger assemblies by reducing intermicellar collision frequency [9].

For pharmaceutical RM systems, especially those integrated into lipid‐based formulations, the hydrophobic phase is not merely a solvent but a functional formulation component, mainly consisting of functional lipids [15]. Medium‐chain triglycerides and mixed lipid systems can impose additional constraints on RM stability and dynamics, necessitating careful co‐optimization of surfactant and this lipid phase to preserve RM integrity upon dilution and digestion [17].

Building on these pharmaceutical requirements, recent next‐generation RM platforms increasingly rely on structured lipid blends to fine‐tune curvature and enhance pP solubilization. A prominent example is the semaglutide RM system, which employs glycerol monocaprylocaprate (Labrafac MC60) and glycerol monolinoleate (Maisine CC) either individually or in defined binary mixtures (1:4, m/m) [21]. These structured lipids exemplify the broader transition from hydrocarbon solvents toward pharmaceutically functional lipid excipients capable of supporting oral biopharmaceutical delivery. Across the RM‐based oral delivery systems summarized in Table 1, several classes of lipid phases appear consistently. Medium‐chain triglycerides, mono‐ and diglyceride systems and mixed lipid excipients dominate due to their favorable balance of fluidity, digestibility and compatibility with surfactant‐driven curvature. More specialized lipid phases—such as isopropyl myristate, salicylic acid methyl ester, or squalane‐based systems—are strategically employed to adjust viscosity, interfacial packing, or payload solubilization.

**TABLE 1:** Composition of reverse micelle systems across reported studies: Continuous hydrophobic phases, surfactant architecture and encapsulated biomolecules.

Continuous hydrophobic phase	Continuous‐phase characteristics	Surfactants	Surfactant content	Encapsulated species	Refs.
Labrafac Lipophile WL 1349	Triglyceride‐rich lipid excipient; biodegradable; medium polarity; low viscosity	AOT	10:1 (w/w)	AP138	[24]
Isooctane	Non‐polar hydrocarbon solvent; highly lipophilic; chemically inert	AOT	0.05 – 0.1M	Aedans HPT Badan Hemoglobin	[25]
Lipoid S 100 dissolved in tert‐butyl alcohol	Phospholipid‐containing organic phase; tert‐butanol provides a polar, water‐miscible solvent environment	Lipoid S 100	50 mg/mL	BSA	[23]
GML (Maisine)	Glycerol monolinoleate‐based lipid; lipophilic amphiphilic glyceride; suitable for lipid‐based formulations	Soybean phospholipid	2 mL: 80 mg	Exe	[26]
Isocetyl stearate n‐octanol MCT (Miglyol 812): Capmul MCM C8 EP/ NF; (1:1, w/w)	Mixed lipid/alcohol phase; MCT and mono‐/diglycerides provide a low‐polarity lipid matrix, while n‐octanol increases phase polarity	Brij 93 Lipoid S 100 Span 80 DDAB	5% 1% DDAB	Methylene blue Safranin O Bromocresol green Methyl orange FL HRP	[18]
MCT: MCM (1:1, w/w)	Triglyceride/mono‐ and diglyceride lipid mixture; biodegradable; low‐to‐medium polarity; suitable for lipid‐based formulations	Tween 85 AOT DODAB Lipoid S 100	10%	Polymyxin B	[17]
Squalene Squalane Liquid paraffin	Highly lipophilic hydrocarbon‐based oils; very low polarity; squalene biodegradable, whereas squalane and paraffin are more chemically inert	AOT Span 80 Lipoid S 100	0.32% to 40% (w/v) 0.1% to 13% (w/v) Lipoid S 100	Ethacridine FL Bacitracin	[27]
Salicylic acid methyl ester and Capmul PG‐2L (1:1, w/w)	Semi‐polar ester‐based organic phase; moderate polarity; suitable for solubilization of amphiphilic pP	Span 80	0.01% to 10% (v/v)	Exe	[28]
IPM Miglyol 812	Fatty acid ester/triglyceride lipid mixture; highly lipophilic; low polarity; biodegradable lipid components	Span 80	2.5 – 3.5%	HRP	[19]
GMCC:caprylic acid (C8) (1:4, m/m) GML: C8 (1:4, m/m) GMCC: GML (1:4, m/m)	Glyceride/fatty acid‐based lipid mixture; amphiphilic; medium polarity; interfacially active, strong interfacial structuring	ELA AOT	0 mM to 300 mM	Semaglutide	[21]
Labrafac Lipophile WL 1349	Triglyceride‐rich lipid excipient; biodegradable; medium polarity; low viscosity	Span 80	5:1 (wt/wt)	Exe	[29]
MCT, (C_8_ 56.5%, C_10_ 43%)	Medium‐chain triglyceride lipid; biodegradable; low polarity; established lipid excipient for oral formulations	Lipoid S 100	—	Insulin	[30]
tert‐butyl alcohol	Polar protic organic solvent; water‐miscible; substantially more polar than conventional hydrocarbon or lipid phases	AOT Lipoid S 100	5%	HRP	[22]
n‐Heptane:benzene	Mixed non‐polar hydrocarbon solvent phase; highly lipophilic; low polarity	BHDC	Not specified; W_S_ = [EG]/[BHDC] varied	Ethylene glycol (EG; non‐aqueous polar phase)	[31]
n‐Heptane	Non‐polar hydrocarbon solvent; highly lipophilic; chemically inert	AOT	Varied polar solvent/AOT ratio	Ethylene glycol (EG), propylene glycol (PG), glycerol (GY), DMF, DMA	[32]
Benzene	Non‐polar aromatic hydrocarbon solvent; highly lipophilic; chemically inert	TX‐100 or BHDC	W_s_ = [IL]/[surfactant] varied	bmimBF_4_ or bmimTf_2_N (ionic‐liquid polar phase)	[33]

As a representative example, Ricci et al. [22] used citronellol and caprylic acid as lipid phase to compare the solubility of native horseradish peroxidase (HRP) with its dRM complex, demonstrating that dRM incorporation markedly increases the protein's affinity for the lipid phase.

In addition to liquid hydrophobic phases, RM‐like architectures have also been reported for solid lipid systems. Chen et al. [23] formulated lecithin‐based solid lipid assemblies by dissolving phosphatidylcholine in tert‐butyl alcohol, yielding so‐called “anhydrous RM” capable of associating hydrophilic proteins such as bovine serum albumin. Although structurally distinct from classical lipid‐based RM, these lecithin assemblies share functional features relevant to early‐stage payload association and therefore represent a solid‐lipid analogue within the broader RM design space. Reported morphological analyses demonstrate the structural diversity observed across RM systems, ranging from classical spherical nanodroplets to more heterogeneous lipid assemblies formed in structured or solid lipid matrices.

These morphological differences become particularly relevant under gastrointestinal conditions, as lipid digestion progresses and triglycerides are enzymatically hydrolyzed into monoglycerides and free fatty acids, resulting in continuous restructuring of colloidal species within the intestinal lumen [15, 34]. The progressive release of free fatty acids and monoglycerides during triglyceride hydrolysis alters the packing parameter and reduces negative interfacial curvature, thereby destabilizing the inverted RM architecture. This shift drives a transition from RM toward mixed monoglyceride/fatty‐acid colloidal phases (e.g., vesicles, mixed micelles), ultimately promoting RM disassembly and facilitating payload transfer into digestion‐derived lipid species [35, 36]. RM‐based architectures are therefore not static entities during gastrointestinal transit, but undergo progressive reorganization driven by lipolysis, bile salt adsorption and dilution [18, 22]. Embedded within the lipids rather than freely dispersed in the aqueous lumen, RM undergo structural rearrangements only as lipolysis progresses, consistent with Cryo‐TEM observations of digestion‐driven transitions in lipid‐colloidal species [37]. These dynamic changes occur later in digestion, whereas the initial interaction of reverse micelles with the surrounding lipid matrix takes place earlier, as reflected by micelle binding to droplet surfaces and early‐phase solubilization phenomena described for lipid‐based formulations [15]. Together, these findings clarify that RM exert their functional effects during the early dispersion phase, before digestion‐induced reorganization dismantles the lipid architecture.

This mechanism is directly supported by the in vivo pharmacokinetic findings of Ebert et al. [19]. While unformulated dRM and wRM exhibit limited bioavailability due to rapid dissociation in the aqueous GI environment, their incorporation into self‐emulsifying inverted lipid phases significantly enhances oral absorption. This experimental evidence confirms that the surrounding lipid vehicle acts as a protective reservoir, enabling reverse micelles to maintain their structural integrity and exert superior solubilization effects prior to full digestion‐driven dismantling.

Given that RM are embedded within a lipid phase that undergoes digestion and emulsification in the gastrointestinal tract, RM are not absorbed as intact nanostructures. Human intraluminal concentration profiling by Brouwers & Augustijns [38] demonstrates that absorption occurs from solubilized molecular species rather than from colloidal entities, meaning that colloidal structures—including RM—do not survive GI transit in a form capable of epithelial uptake. This implies that RM are progressively dismantled as lipolysis and bile salt–mediated emulsification proceed. Consequently, the functional role of RM lies in the early stages of dispersion and digestion, where they facilitate initial association of hydrophilic payloads with the lipid phase and stabilize them before digestion‐driven disassembly occurs [17, 18, 22].

This behavior is strongly governed by the intrinsic polarity of the hydrophobic phase, which governs its capacity to solubilize water. More polar lipids, such as medium‐chain triglycerides, exhibit a higher water uptake and enable the formation of aqueous domains, whereas nonpolar oils like isopropyl myristate show only minimal water incorporation [19]. In contrast, highly apolar hydrocarbons such as squalane lack sufficient polarity to solubilize water at all, thereby preventing the formation of reverse micellar structures and highlighting the critical role of oil polarity in dRM formation and functionality [8].

Building on this foundation, structure‐specific RM mechanics must be clearly separated from non‐specific excipient effects, such as general membrane permeation enhancement or enzyme inhibition [39]. Unstructured surfactant–lipid mixtures lacking an ordered RM architecture cannot form a thermodynamically stable polar core within the lipophilic matrix [40, 41]. Consequently, hydrophilic payloads fail to undergo molecular solubilization and remain unprotected against luminal enzymatic cleavage [42]. In contrast, the formation of an ordered RM architecture—governed by a critical packing parameter (*N_s_* > 1) and defined interfacial curvature—creates a localized nanodomain that physically sequesters the hydrophilic payload [43]. This structural sequestration provides essential steric shielding, establishing a mechanistic feature that cannot be replicated by unstructured excipient mixtures alone.

### 2.2 Surfactants

#### 2.2.1 Headgroup Chemistry

The formation, stability and structural characteristics of RM are governed primarily by the molecular architecture of the surfactant, including headgroup chemistry, hydrophobic tail length and overall molecular geometry [44]. Ionic surfactants such as sodium bis(2‐ethylhexyl) sulfosuccinate (AOT) have historically dominated RM research due to their strong propensity to form stable RM across a wide range of water loadings [6, 45, 46]. However, non‐ionic and zwitterionic surfactants have increasingly attracted attention owing to their reduced interfacial charge density and improved biocompatibility, particularly in formulations intended for pharmaceutical or biological use [10, 47, 48]. Cationic surfactants can strongly interact with cellular membranes, but their positive charge is frequently associated with elevated cytotoxicity and membrane‐disruptive effects, which restricts their applicability in oral pP delivery [49]. Biodegradable ester‐linked surfactants such as ethyl lauroyl arginate (ELA) provide a more biocompatible alternative, enabling RM formation without the safety liabilities inherent to, in particular, permanently positively charged surfactants. Sandmeier et al. [21] demonstrated this by successfully employing ELA as a low‐toxicity surfactant in RM‐based pP formulations.

Beyond this compositional diversity, the interface of an RM—defined by the surfactant headgroups—is a central determinant of RM properties [6]. The chemical identity of the headgroup (anionic, cationic, zwitterionic or non‐ionic) controls interfacial polarity, electrostatics and hydrogen‐bonding networks within the inner core, directly influencing the solubilization capacity and dynamics of encapsulated species.

#### 2.2.2 Packing Geometry

Surfactant packing behavior can be rationalized using geometric packing concepts, where the balance between headgroup area and tail volume dictates the preferred curvature of the self‐assembled interface [44]. RM require high negative curvature, and even subtle structural modifications—such as branching of hydrophobic chains or changes in headgroup hydration—can lead to pronounced shifts in RM size, shape and stability [10, 50, 51]. These effects are amplified at low water contents, where interfacial forces dominate over bulk‐like core properties [6, 8].

Surfactant architecture dictates interfacial curvature and packing, which in turn governs RM size and shape [8, 44, 50]. The packing preference is frequently described by surfactant geometric parameters (see Figure 2). Variations in headgroup area, tail volume or the presence of cosurfactants modify curvature strain and thereby enable fine‐tuning of RM structure for specific applications [47, 52].

**FIGURE 2:**
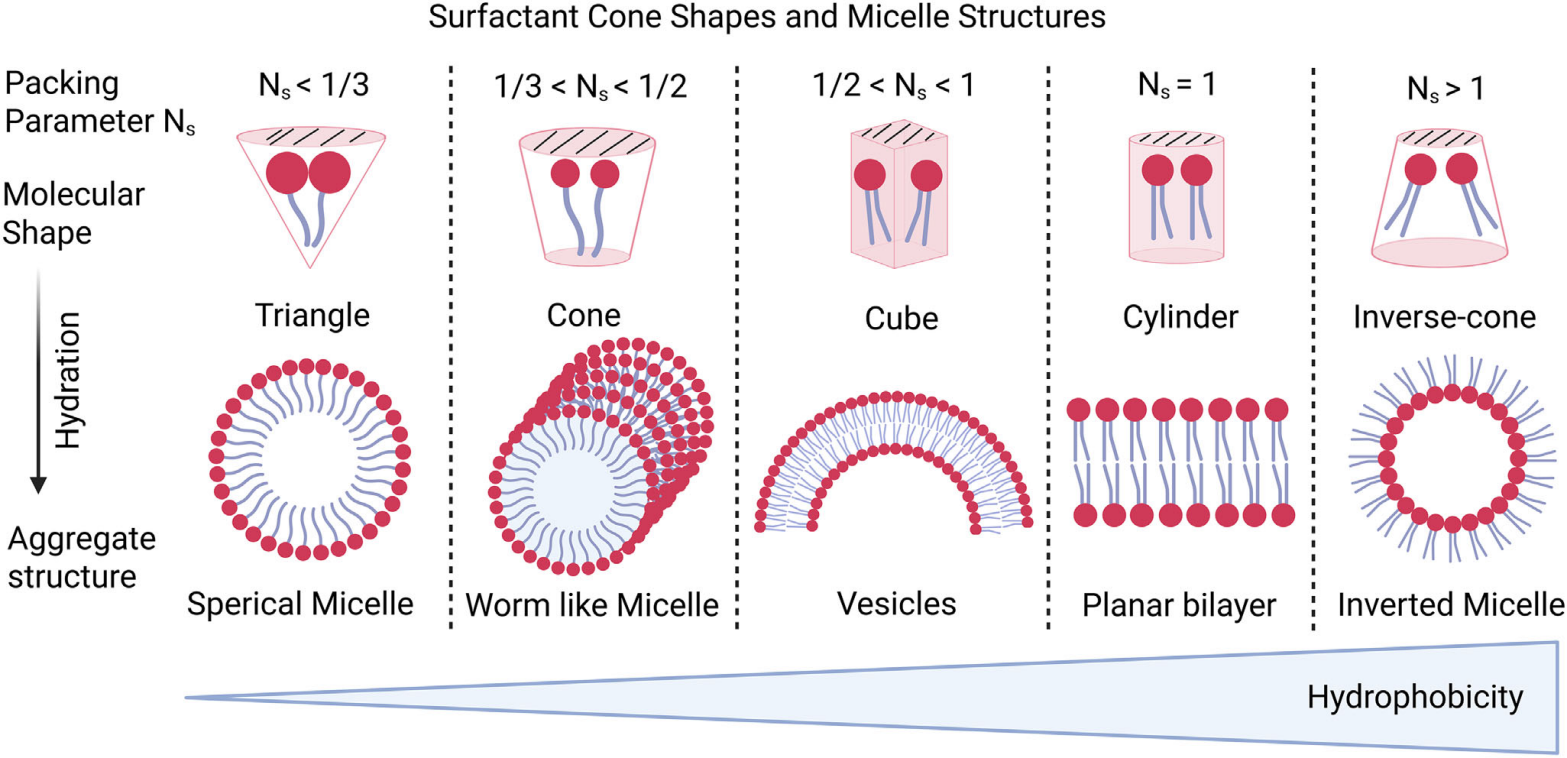
Relationship between surfactant geometry, packing parameter (Ns), self‐assembly and hydrophobicity.

Such curvature‐dependent behavior is mirrored in the selection of surfactants used in contemporary RM formulations. Non‐ionic surfactants such as sorbitan monooleate (Span 80) and polyoxyethylen‐sorbitan‐trioleat (Tween 85) are widely used due to their mild interfacial properties and broad formulation compatibility, with Span 80 favoring the formation of water‐in‐oil structures and Tween‐type polysorbates providing solubilization and emulsification capacity in complex mixtures. Schmidt et al. [49] showed that zwitterionic phosphatidylcholine‐based excipients (e.g., Lipoid S100) enhance biocompatibility by offering membrane like interfacial characteristics that reduce surfactant related toxicity. Together, these surfactant classes define the interfacial design space from which contemporary RM architectures are constructed.

As illustrated in Figure 3, the effective cone geometry of a surfactant—ranging from triangular and conical shapes to more block‐like, cylindrical or inverted‐cone structures—directly dictates the curvature of the interface and therefore the size of the resulting RM. Surfactants with larger packing parameters adopt more lipophilic, inverted‐cone geometries that enforce high curvature and generate small aqueous cores, whereas more cylindrical shapes allow the formation of larger, less curved water domains such as vesicles or planar bilayers. Because the achievable core volume ultimately sets the upper limit for solubilization, both packing geometry and the critical micelle concentration (CMC) indirectly correlate with payload capacity by influencing assembly number and accessible hydration. Since payload capacity scales with the available aqueous volume, any structural feature that restricts curvature or hydration inherently limits how much pP can be solubilized [43, 47].

**FIGURE 3:**
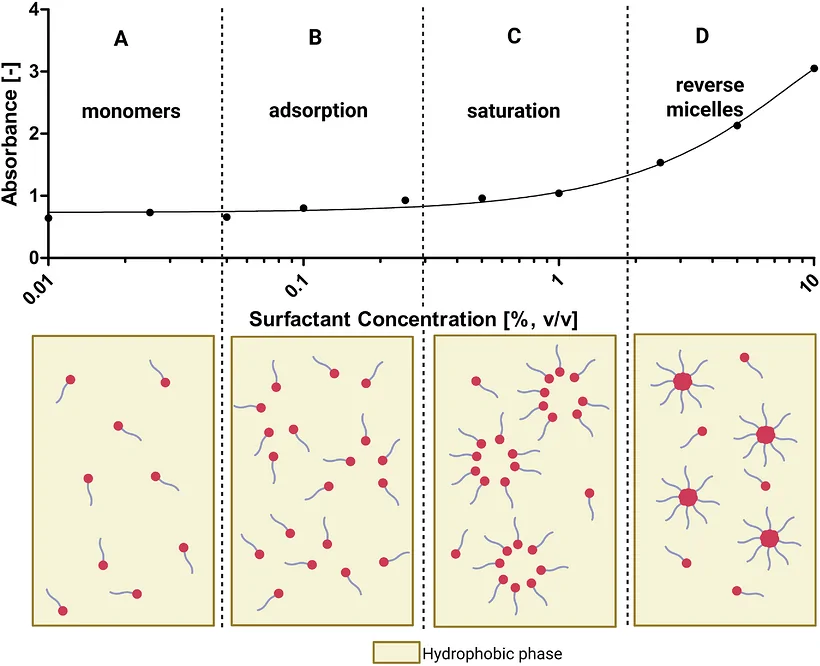
Concentration‐dependent behavior of sorbitan monooleate in the hydrophobic phase; from sub‐CMC monomers to reverse micelle formation.

Pires et al. [53] emphasize that the diameter of the micellar core is not governed by surfactant concentration alone. Instead, surfactant‐specific packing characteristics—including headgroup area, tail volume, spontaneous curvature and the resulting assembly number—impose geometric constraints that define the maximum attainable core size at a given hydration level. Consequently, even at identical water loadings, different surfactants generate markedly different core diameters due to their intrinsic packing properties. Representative examples of pP successfully incorporated into RM using different surfactant systems illustrate this breadth: large proteins such as albumin (∼65 kDa) can be incorporated using AOT, whereas small proteins like rubredoxin (∼5 kDa) associate efficiently with trioctylmethylammonium chloride (TOMAC). Even proteins with extreme isoelectric points, such as lysozyme (pI 11.0), can be solubilized in RM formed by AOT or TOMAC. These examples highlight how surfactant packing geometry, rather than concentration, dictates the accessible core size and thereby the solubilization range of different pPs.

#### 2.2.3 Critical Micelle Concentration and Self‐Association Behavior

Beyond interfacial curvature and excipient compatibility, the ability of a surfactant to form RM is strongly influenced by its CMC and its capacity for water uptake. Micellization occurs once the concentration of monomeric surfactant exceeds the CMC, at which point the system minimizes its free energy by reducing the contact area between hydrophobic tails and the continuous phase [43, 47]. This process proceeds through a sequence of steps: accumulation of monomers at the interface, saturation of interfacial sites and subsequent formation of nanoscale assemblies.

Surfactants with low CMC values, such as polysorbates and phosphatidylcholine‐based lipids, can therefore form associates at very low concentrations, which is advantageous in formulations where interfacial charge must be minimized. In contrast, sorbitan esters like Span 80 exhibit extremely low CMCs but limited water uptake, restricting the maximum achievable w_0_ while favoring highly curved w/o structures [54, 55].

Figure 3 exemplifies the organization of Span 80 in the hydrophobic phase, showing the transition from dispersed monomers (A) to adsorption at water droplets (B), interfacial saturation (C) and the formation of closed reverse‐micellar shells (D). This sequence aligns with previous descriptions of Span 80 behavior in nonpolar media [54, 55].

#### 2.2.4 Dynamic Nature of RM: Fusion‐Fission and Molecular Exchange

These hydration‐dependent changes in internal structure also modulate the dynamic behavior of RM, including fusion–fission events and intermicellar exchange. Surfactant monomers and encapsulated molecules can migrate between individual RM through transient fusion–fission events. In these events, two RM collide, form a short‐lived dimer or “necked” intermediate and subsequently separate, redistributing both surfactant and internal contents [9, 56, 57, 58]. Time‐resolved spectroscopic and NMR studies have shown that these encounters occur on micro‐ to millisecond timescales and can enable rapid equilibration of solutes between RM, even when individual assemblies remain nanoscale and discrete [56, 58, 59, 60]. In AOT‐based systems, for example, exchange of small solutes and ions has been directly correlated with collision frequency and the lifetime of fusion intermediates, highlighting the role of intermicellar interactions in governing transport processes [8, 56, 57]. These mechanistic steps are summarized in Figure 4, which depicts the fusion‐fission cycle and the transient dimeric intermediates that mediate intermicellar exchange of water, surfactant monomers and encapsulated solutes.

**FIGURE 4:**
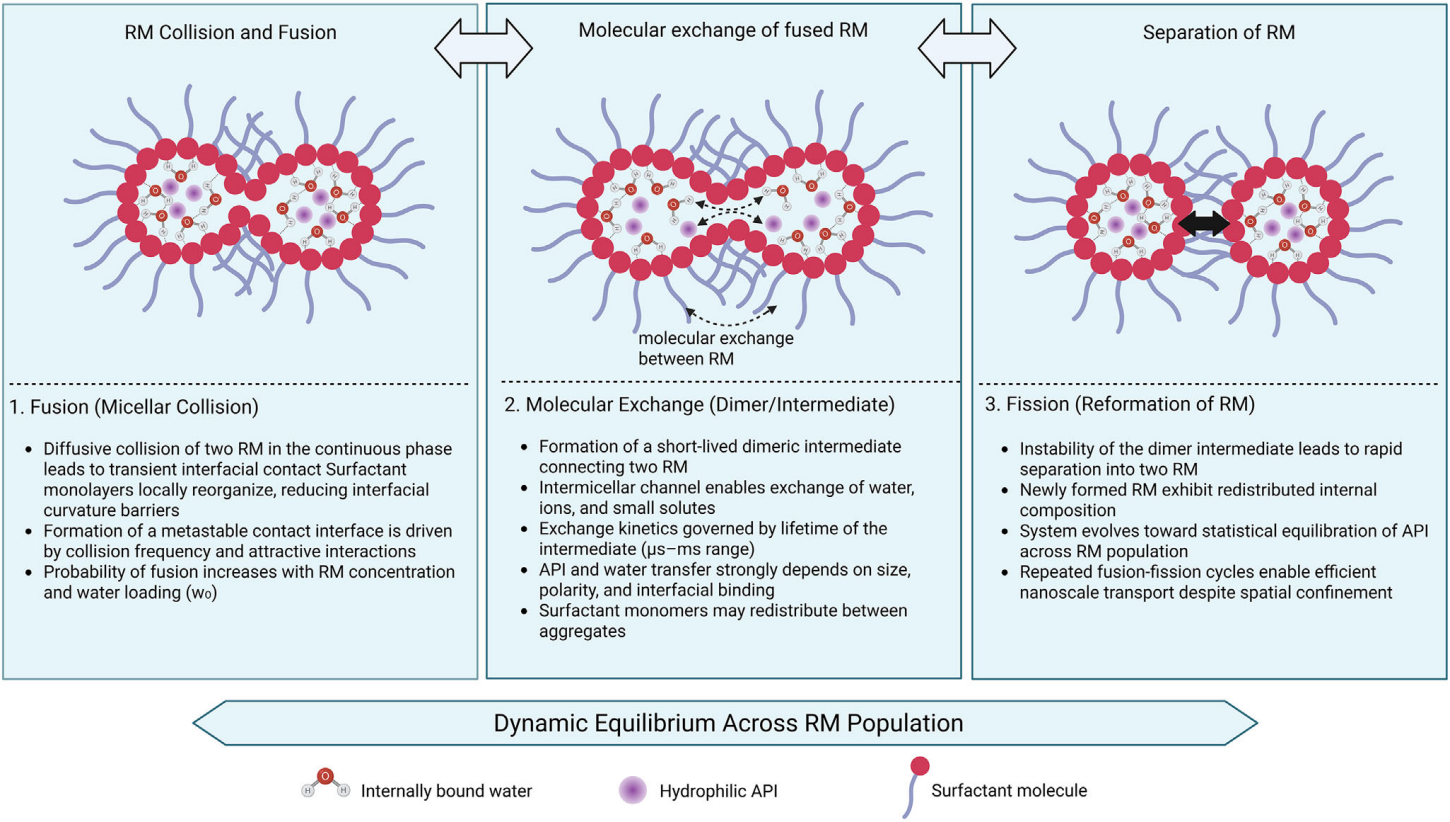
1. Fusion: diffusive collision of two reverse micelles leading to transient interfacial contact. 2. Molecular exchange: formation of a short‐lived dimeric intermediate enabling transfer of water, surfactant monomers and solutes and 3. Fission: separation into two reverse micelles with redistributed internal composition.

The frequency and extent of these dynamic exchanges are influenced by RM composition, water loading, surfactant type and continuous phase properties [7, 61, 62]. Higher w_0_ values typically correlate with increased exchange dynamics, whereas systems with very low water content or strongly bound interfacial layers exhibit slower dynamics. These dynamic processes directly impact solute partitioning, encapsulation stability and catalytic turnover in RM media, as the rate of intermicellar exchange can become rate‐limiting for reactions involving spatially separated reactants [56, 57, 58, 59].

In systems containing structured or partially solidified hydrophobic phases, these fusion–fission events are markedly suppressed, as the reduced interfacial fluidity and restricted surfactant mobility prevent the formation of transient dimeric intermediates. As a result, intermicellar exchange becomes strongly attenuated, and solute transport is dominated by intradroplet processes rather than dynamic collisions, consistent with the solid‐like, low‐mobility lecithin inversion structures reported by Chen et al. [23].

### 2.3 Non‐Aqueous and Ionic‐Liquid Reverse Micelles

Although RM are most commonly discussed in the context of water‐in‐oil systems, their structural diversity extends beyond aqueous cores and conventional non‐polar continuous phases. For example, AOT‐based RM have been described in n‐heptane containing non‐aqueous polar solvents such as ethylene glycol, glycerol, and dimethylformamide (DMF), where interactions between the polar solvent and the surfactant headgroups contribute to the organization of the RM interface and the properties of the confined phase [32, 63]. Similarly, non‐aqueous RM containing ethylene glycol have been formed using benzene/n‐heptane mixtures as the external phase, demonstrating that stable reverse‐micellar structures can be established with polar solvents other than water and mixed non‐polar continuous phases [31]. Ionic liquids provide a further extension of this concept. Reverse micelles have been generated using ionic liquids as confined polar phases, including ionic‐liquid/surfactant/benzene systems in which the structure and microenvironment of the confined phase depend strongly on the surfactant chemistry [33]. Conversely, AOT‐based RM containing water domains have also been formed within hydrophobic room‐temperature ionic liquids, further demonstrating the structural versatility of RM in ionic‐liquid environments [64]. Collectively, these systems highlight that RM should be regarded as a broader class of self‐assembled interfacial environments rather than exclusively water‐containing structures, in which the nature and localization of the polar phase can be modulated through solvent, surfactant, and ionic composition. For oral pP delivery, however, the present review focuses on RM architectures compatible with biocompatible lipid‐based formulations, while these broader systems provide important mechanistic context for understanding interfacial organization and ionic interactions.

### 2.4 Co‐Surfactants

Co‐surfactants such as short‐chain alcohols or amphiphilic co‐lipids are frequently employed to modulate interfacial properties in RM systems [10, 65]. By inserting into the surfactant layer, co‐surfactants can reduce interfacial tension, relieve curvature stress and broaden the stability window of RM systems [10, 65]. This approach is particularly useful for tailoring RM properties when working with bulky or sensitive payloads.

Beyond classical co‐surfactants, more advanced RM formulations incorporate functional additives to fine‐tune interfacial composition, microenvironment polarity and payload–surfactant interactions. Charged species—including cationic surfactants such as dimethyldioctadecylammonium bromide and didecyldimethylammonium bromide– are used when electrostatic interactions with pP payloads provide a stabilizing or solubilizing advantage [17, 18]. Emerging systems increasingly employ functional surfactants such as ethyl lauroyl arginate to modulate interfacial charge and enhance pP solubilization, reflecting a shift toward more tailored interfacial engineering [21]. In contrast to such functional additives, anionic surfactants such as sodium docusate remain central to RM formation due to their strong curvature‐inducing capacity and their ability to reliably stabilize water‐in‐oil nanodomains across a wide range of compositions [17, 21, 22, 27].

### 2.5 Additives

Additional additive classes, including polymers, co‐solvents and complexing agents, further expand the design space of RM formulations [66]. These components can adjust viscosity, alter headgroup spacing, stabilize transient structures or influence rehydration behavior. While such strategies offer enhanced control over RM architecture and performance, they also increase system complexity and can significantly affect RM dynamics and rehydration behavior—effects that are particularly pronounced in dRM systems [9, 22, 46].

In addition to interfacial modifiers, certain additives are introduced during processing to facilitate drug incorporation. Lindner et al. demonstrated that hydrophilic payloads can be pre‐dissolved in volatile organic co‐solvents such as ethanol or 2‐propanol, which are subsequently evaporated to yield drug‐loaded dry RM. These co‐solvents act as transient processing aids rather than formulation components, but they can strongly influence payload incorporation efficiency and the resulting RM microenvironment [27, 67, 68].

In line with their role as interfacial modifiers and processing aids, hydrophilic organic solvents can also directly influence water distribution and transport in RM systems. By partitioning into the interfacial region and/or the confined polar phase, these solvents modify local polarity, hydrogen‐bonding networks and chemical potential gradients, thereby affecting water mobility and exchange processes [69]. Correa et al. systematically demonstrated that polar organic solvents such as methanol, acetonitrile or dimethyl sulfoxide (DMSO) do not simply replace water but create distinct microenvironments within RM, characterized by altered hydrogen‐bonding networks and polarity profiles. Their work showed that these solvents can partially substitute or reorganize interfacial water, leading to changes in solute solubilization, mobility and exchange behavior. In particular, the authors highlighted that the presence of such solvents modifies the balance between interfacial and core‐associated water, thereby directly influencing water dynamics and transport processes within and between RM [69].

Short‐chain alcohols, such as ethanol, preferentially localize at the interface, where they reduce headgroup packing and increase interfacial flexibility. As a result, water exchange is facilitated both between individual RM via fusion–fission processes and across the hydrophobic phase‐aqueous phase boundary [70, 71]. In contrast, highly polar aprotic solvents such as DMSO more strongly alter the internal polarity of the RM core, which can disrupt hydrogen‐bonding networks and influence water retention and mobility [69, 72].

Thus, beyond their function in payload solubilization or processing, hydrophilic solvents act as active modulators of RM water dynamics. While they can promote water uptake and redistribution, they may simultaneously reduce interfacial rigidity and thereby impact RM stability, particularly in more hydrated systems.

### 2.6 Active Pharmaceutical Ingredients

Encapsulation of hydrophilic active pharmaceutical ingredients (API) in RM is governed by two principal mechanisms: solubilization within an internal aqueous core or interfacial association with the surfactant layer. In conventional RM containing a discrete water pool, APIs are predominantly solubilized within the confined aqueous domain [73]. In contrast, water‐deprived systems lack a continuous aqueous phase, and stabilization therefore occurs predominantly at the surfactant–oil interface through a combination of electrostatic interactions, hydrogen bonding, hydration effects, and, depending on the surfactant and API, ion pairing with surfactant headgroups [6, 22, 46]. Importantly, the contribution of electrostatic interactions in RM should not be equated with classical hydrophobic ion pairing (HIP). Whereas HIP generally involves the formation of a defined, charge‐dependent API–counterion complex to increase the lipophilicity of the drug, RM provide an organized interfacial environment in which multiple surfactant molecules can interact simultaneously with different polar or charged regions of the API. Thus, ionic interactions may contribute substantially to RM stabilization without constituting the sole driving force for incorporation or requiring a stoichiometric ion pair. Accordingly, wet RM (wRM) are characterized by the presence of a discrete internal aqueous phase, whereas dRM contain only residual water (≤1% w/w) [18], below which bulk‐like water is absent and stabilization becomes predominantly interfacial, as discussed in Section 3.

Consequently, loading efficiency is determined by the balance between aqueous solubility and interfacial affinity, which are both highly sensitive to the API charge state, polarity and hydrogen‐bonding capacity. Even subtle structural variations can therefore lead to pronounced differences in localization and retention within the near‐anhydrous interfacial environment [6, 9, 46, 58].

pP exhibit strong interfacial association within RM but are prone to interfacial denaturation in highly dynamic or strongly charged environments [6, 21]. In dRM, reduced water activity and restricted molecular mobility stabilize pP by limiting conformational fluctuations, unfolding and aggregation, while preserving localized hydration layers required for structural integrity [17, 18, 19, 46]. Although this interfacial mode of association may constrain maximum loading, it supports enhanced storage stability and retained biological activity. Upon emulsification in gastrointestinal fluids, these systems still enable controlled release, linking interfacial confinement to functional performance [22, 46, 56].

The successful incorporation of hydrophilic API into RM strongly depends on the chosen loading strategy. In dRM systems in particular, encapsulation is inherently process‐driven rather than purely composition‐dependent, making the preparation route essential for achieving high loading, retention and overall formulation performance.

### 2.7 Dry–Addition Process Route—Direct Incorporation into Precursor Assemblies

In the dry‐addition (DA) approach, the hydrophilic payload is introduced directly, typically as a solid, into a lipophilic phase containing RM‐forming surfactants [17]. This solvent‐free method is operationally simple but relies on efficient wetting, deagglomeration and interfacial transfer into the surfactant headgroup region (Figure 5A). While effective for selected pP‐surfactant systems, DA is often limited by mass transfer barriers and incomplete incorporation, particularly for poorly wettable or cohesive solids, leading to heterogeneous systems with variable performance [18, 27].

**FIGURE 5:**
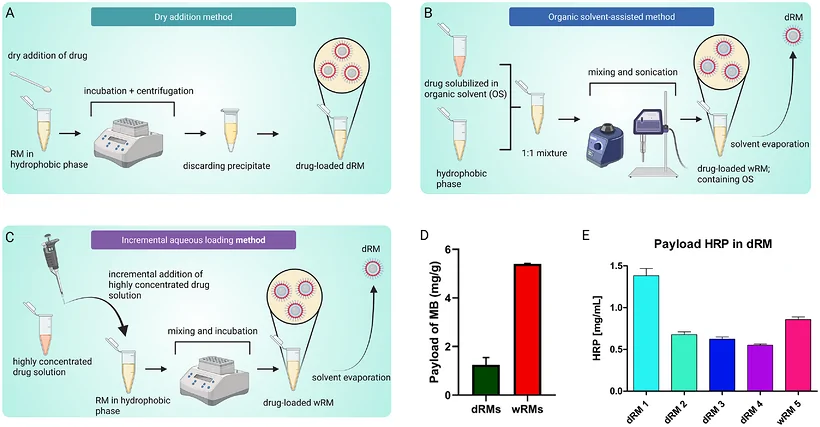
Process routes for the preparation of dry reverse micelles (dRM) and their impact on payload incorporation. (A) Schematic representation of the dry‐addition (DA) method. (B) Organic solvent–assisted (OS) method. (C) Incremental aqueous loading approach. (D) Quantitative comparison of payload incorporation for methylene blue. Reproduced from Eczacioglu et al. [18] under the terms of the Creative Commons CC BY license. (E) Effect of preparation method on apparent solubilization capacity.Reproduced from Ebert et al. [19] under the terms of the Creative Commons CC BY license.

### 2.8 Organic Solvent‐Assisted Process Route

In the organic solvent‐assisted (OS) route, the payload is first dissolved or dispersed in a volatile polar solvent (e.g., ethanol, 2‐propanol) and subsequently mixed with the surfactant‐containing lipophilic phase (Figure 5B,E) [27]. Controlled solvent removal yields dRM with a more homogeneous interfacial payload distribution. This approach enhances loading by increasing local payload concentration at the interface prior to dehydration, thereby overcoming mass transfer limitations, particularly for poorly wettable compounds [6, 8, 18, 74]. Solvent removal is typically achieved by vacuum centrifugation or rotary evaporation, requiring precise control to prevent phase separation or aggregation [7, 22, 27, 75] Comparative studies consistently show improved apparent solubilization and retention compared to DA, attributed to more uniform molecular dispersion prior to drying [22, 27].

### 2.9 Incremental Aqueous Loading Method

An alternative strategy, described by Ricci et al. [22], combines stepwise aqueous loading with controlled dehydration (Figure 5C,D). Here, the pP is dissolved in an aqueous phase and added incrementally to a pre‐formed lipophilic surfactant system, enabling gradual incorporation into the headgroup region while maintaining dispersion stability. The system transiently adopts a hydrated state, with loading limited by turbidity or phase instability. Subsequent dehydration, e.g. via vacuum centrifugation, converts the system into RM while preserving interfacial organization and minimizing stress on the biomolecule. By separating loading and drying, this method enables homogeneous distribution and effectively “locks” the payload within the interface, resulting in improved retention, preserved enzymatic activity and favorable redispersion behavior. Mixed surfactant systems further allow tuning of interfacial properties and compatibility with lipid‐based delivery systems [17, 22].

## 3 Role of Water in RM

### 3.1 Bulk‐Like Water and Surfactant‐Bound Water

Water within RM exists in structural states that differ fundamentally from bulk water. At low water loadings (small values of w_0_, defined as the molar ratio of water to surfactant), water molecules are strongly bound to surfactant headgroups, forming tightly coordinated hydration shells with reduced translational freedom [46, 47, 73, 76]. As w_0_ increases, RM cores expand and internal water progressively assumes bulk‐like properties, including higher mobility, extended hydrogen bond networks and reduced interfacial influence [77]. SANS, SAXS, NMR and MD studies consistently demonstrate how increasing water content leads to more disordered water and larger polar domains [9, 78, 79, 80]. Exemplary determination of water content in RM formulations can be achieved via the water signal at 3.33 ppm in ^1^H‐NMR, as demonstrated by Eczacioglu et al. and Ebert et al. [18, 19]. Determining the water content—and thus distinguishing wet from dry RM—is critical for predicting the stability of encapsulated pP, since conformational flexibility and reaction kinetics are highly sensitive to water structure. Accordingly, the ratio of surfactant‐bound to bulk‐like water represents a key determinant of functional behavior in RM‐based systems [6, 73, 81].

The molar water‐to‐surfactant ratio w_0_ thus serves as a central structural and functional control parameter. It correlates directly with the size of the internal polar domain [45, 76, 77], with low w_0_ values dominated by bound water and higher values leading to the emergence of internal water‐pools [58, 82]. Numerous SAXS, SANS and NMR studies report a quasi‐linear relationship between w_0_ and RM radius in classical systems such as AOT‐based RM [45, 76, 77]. Functionally, w_0_ governs RM dynamics, solute mobility and biochemical activity, including enzymatic reactions and pP stability [9]. In dRM, w_0_ approaches zero in the classical sense, eliminating bulk‐like water and shifting the dominant stabilizing forces from water‐mediated interactions to surfactant–solute and surfactant–surfactant interactions [18, 46, 83]. This shift defines the fundamental boundary between wet and dRM and explains their divergent structural and functional behavior.

A detailed experimental approach to characterize water structure and dynamics in RM was reported by Roche et al. [25], who investigated hydration behavior in hemoglobin‐loaded RM using fluorescence‐based probes and controlled osmotic dehydration (Figure 7). Their work provides direct insight into the distribution and mobility of water populations as a function of hydration level, thereby complementing the structural description introduced above. To probe water mobility, the authors employed environment‐sensitive fluorophores, including pyranine and covalently attached Badan. Pyranine exhibits two emission bands whose intensity ratio reflects the mobility of surrounding water molecules, distinguishing mobile, bulk‐like water from restricted, interfacial water [25]. This enables a sensitive, semi‐quantitative assessment of the relative contributions of bulk‐like and surfactant‐bound water populations within the RM core.

Water content was systematically tuned using polyethylene glycol (PEG) as an osmotic stress agent, which gradually withdraws water from pre‐formed RM systems [25]. In the context of the w_0_ concept, this corresponds to a controlled reduction of hydration, allowing observation of the transition from high to low w_0_ states. The results show that decreasing water content reduces mobile water fractions and increases strongly bound, interfacial hydration layers, accompanied by changes in pP dynamics consistent with “solvent slaving” [25]. This confirms that the transition from bulk‐like to surfactant‐bound water occurs progressively with decreasing hydration, in line with the w_0_‐dependent structural evolution described above. From a formulation perspective, these findings support the distinction between hydration regimes: systems at higher effective hydration (high w_0_) contain significant fractions of mobile, bulk‐like water, whereas low‐w_0_ systems are dominated by immobilized, surfactant‐bound hydration layers. Water mobility therefore emerges as a key determinant of RM structure and function beyond total water content.

Figure 6 illustrates the structural differences between wRM and dRM, highlighting the presence of a bulk‐like aqueous core in wRM and the exclusively interfacial, surfactant‐bound hydration in dRM. wRM contain a discrete internal polar domain whose size and dynamics are governed by w_0_, which determines micellar size, interfacial curvature, internal mobility and solute transport [6, 77]. Their aqueous cores span a gradient from strongly interfacial water at low w_0_ to increasingly bulk‐like water at higher values [46, 58]. Bulk‐like water exhibits higher translational mobility and extended hydrogen‐bond networks, whereas surfactant‐bound water remains highly structured and interfacially constrained [9, 20, 61, 62, 84].

**FIGURE 6:**
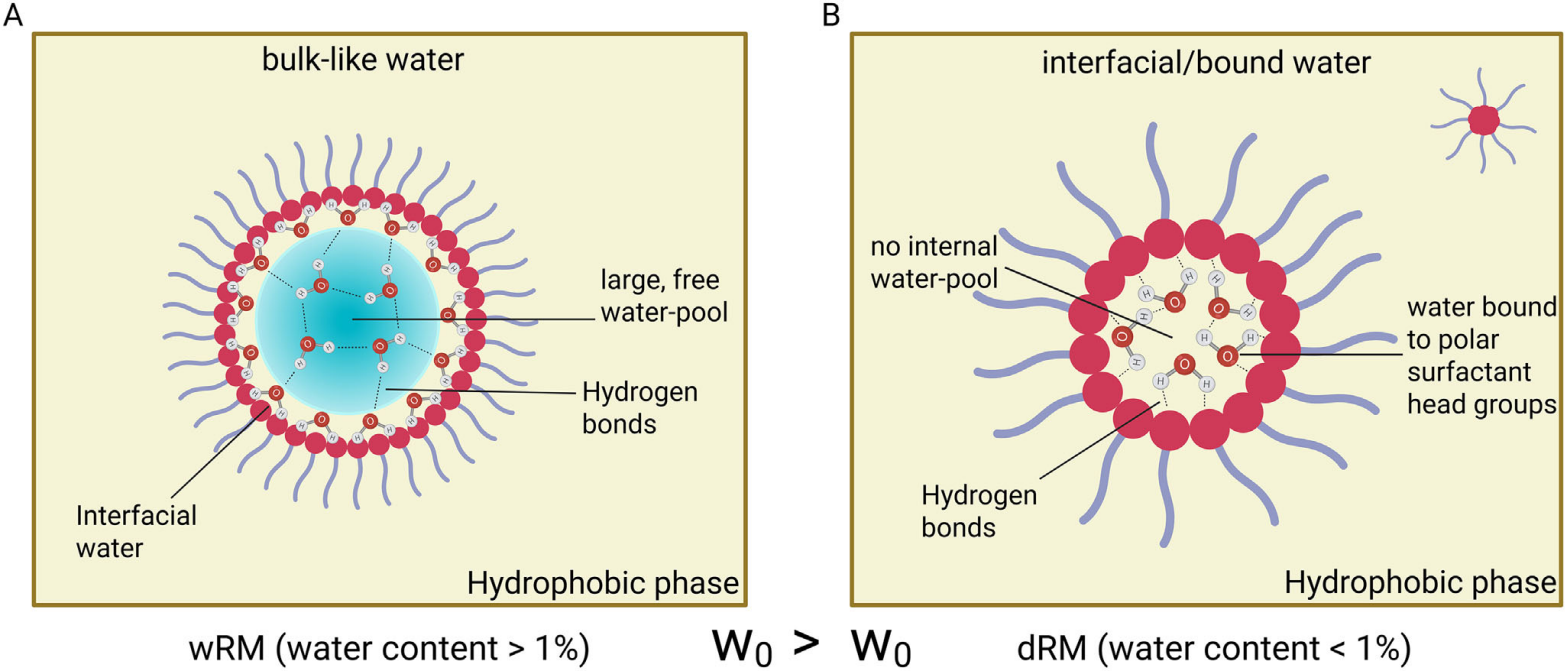
Hydration states in wet and dry reverse micelles (wRM vs dRM), comparison of bulk‐like water in wRM and bound interfacial water in dRM.

In contrast, dRM lack a continuous bulk‐like aqueous phase but retain a confined polar microenvironment capable of stabilizing hydrophilic or charged species, including pP [17, 18, 83]. Here, polar components associate primarily with surfactant headgroups or exist as tightly bound hydration layers rather than forming a continuous water pool [22, 46, 84]. dRM are therefore not dehydrated wRM but distinct supramolecular assemblies stabilized predominantly by interfacial interactions rather than internal water structure [6, 46, 74, 85, 86, 87]. As a consequence of the absence of bulk‐like water, osmotic pressure is reduced and fusion–fission dynamics are suppressed, leading to kinetically stabilized nanostructures with reduced internal mobility. This altered dynamic regime contributes directly to the enhanced storage stability of dRM compared to wRM [18, 19, 46, 56].

### 3.2 Water Uptake Capacity

Water uptake studies are widely used to probe the capacity of RM systems to incorporate and accommodate polar phases, providing indirect insight into internal structure, interfacial flexibility and formulation robustness. Mechanistically, water uptake in wRM involves progressive incorporation of water molecules into the polar core, resulting in micellar swelling, reorganization of the surfactant interface and a gradual transition from strongly bound interfacial water to increasingly bulk‐like water domains. This process is enabled by the elasticity of the surfactant film and its ability to adjust curvature in response to hydration. In contrast, dRM lack a pre‐existing aqueous core; water uptake is therefore largely restricted to hydration of surfactant headgroups and formation of tightly bound interfacial water layers, with only limited structural expansion.

This compositional dependence is clearly illustrated in Figure 7. Panel A shows that water uptake increases systematically with both rising surfactant (Span 80) concentration and increasing hydrophilicity of the continuous phase. Formulations containing higher proportions of MCT exhibit substantially greater water uptake and fall within the wRM regime, whereas IPM‐rich systems remain confined to low water uptake and correspond to dRM. The sharp transition between these regimes highlights how formulation polarity governs the ability of RM to accommodate aqueous domains. Panel B visualizes the structural consequences of these differences: wRM with high water uptake form enlarged internal water pools and undergo micellar swelling, resulting in clear dispersions, whereas dRM only form tightly bound interfacial hydration layers, maintain small diameters and appear turbid.

**FIGURE 7:**
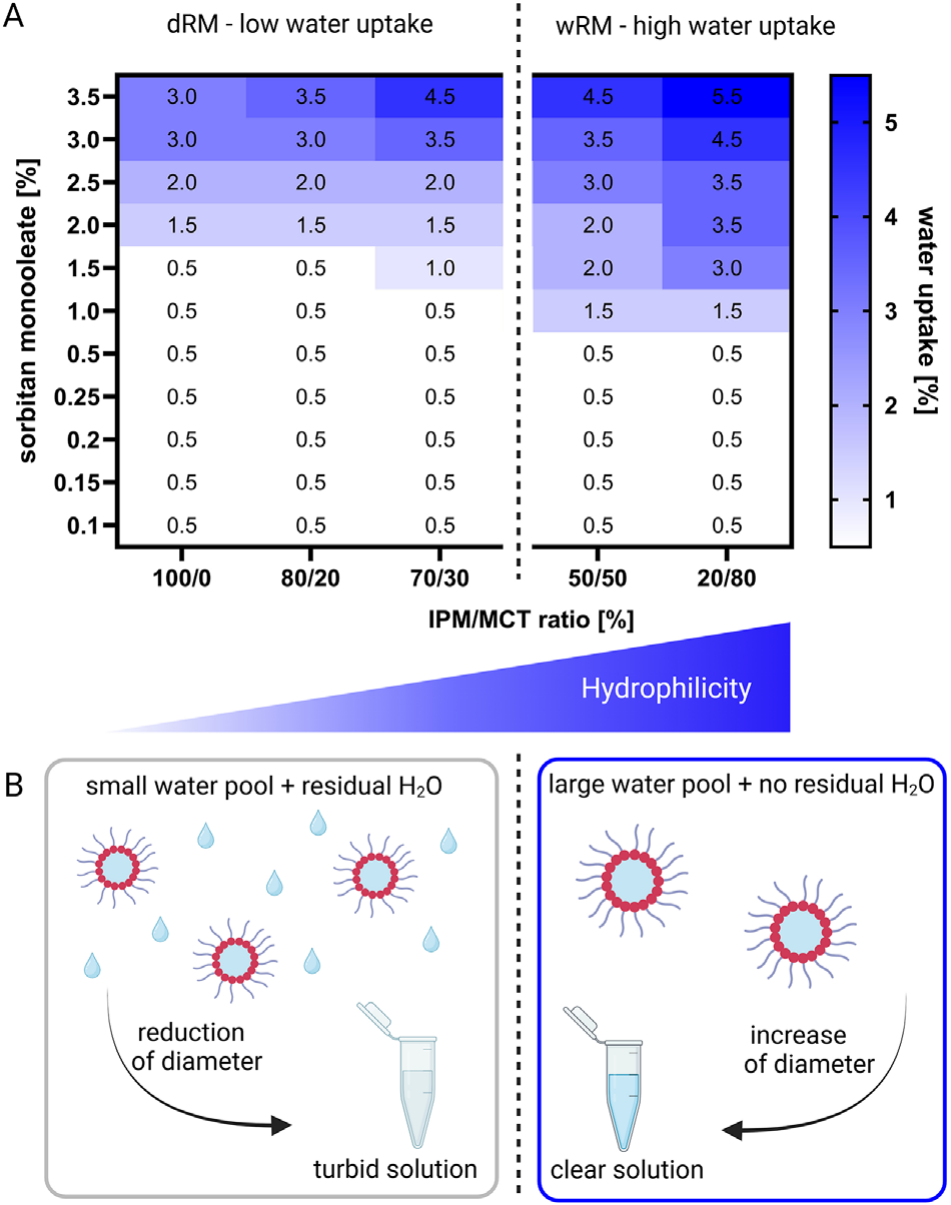
(A) Heatmap of maximum water uptake across IPM/MCT formulations containing 0.1%–3.5% sorbitan monooleate. (B) Schematic illustration of water uptake behavior in dry versus wet reverse micelles. Data from Ebert et al. [19].

A rising number of experimental findings demonstrates that the extent of water uptake is strongly dependent on formulation composition and polarity. In particular, studies by Jörgensen et al. [17] on lipid‐based RM systems confirmed that formulations containing more hydrophilic lipid components exhibit enhanced water uptake due to increased affinity for polar species and greater interfacial adaptability. Similarly, Ebert et al. [19] showed that inverted lipid phases and RM‐based systems with higher polarity display increased water uptake and structural rearrangement upon exposure to aqueous media. Complementary findings by Eczacıoglu et al. [18] who directly compared dry and wet RM in SEDDS, confirmed that classical wRM systems generally exhibit significantly higher water uptake than dRM, particularly when composed of more hydrophilic excipients. In contrast, dRM remain comparatively resistant to water uptake, reflecting their stabilization through interfacial interactions rather than bulk aqueous domains. As a result, water uptake experiments provide a useful mechanistic discriminator between wRM and dRM systems and offer insight into their expected behavior under dilution and gastrointestinal conditions [18, 19].

### 3.3 Structural and Functional Distinction Between wRM and dRM

wRM contain an internal aqueous domain that shapes their structure, dynamics, and solute mobility [6]. In terms of hydration limits, wRM represent an intermediate transition regime (*W*_0_ ≈ 5‐15) situated below fully swollen w/o microemulsions (*W*_0_ > 15). According to the spectroscopic core/shell model established by Fayer & Levinger [88], encapsulated water partitions into an interfacial shell bound to the surfactant headgroups and an inner core ensemble. In the wRM regime (*W*_0_ ≲ 15), structural perturbations from the charged interface extend throughout the entire aqueous cavity; consequently, core water molecules remain strictly nanoconfined and dynamically perturbed, lacking the rotational relaxation properties of true bulk water. Only as hydration increases beyond *W*_0_ ≈ 16.5 (droplet core diameters ≥ 5.5 nm) does a central water ensemble recover unperturbed bulk‐like properties—a threshold that defines the onset of fully developed w/o microemulsions. With decreasing water content (*W*_0_), the system progressively transitions toward dRM, accompanied by a fundamental shift in stabilization from water‐containing to interface‐dominated assemblies [18, 22, 46].

Mixed reverse micellar assemblies also occur, including wet mixed RM with a continuous aqueous core and dry mixed RM in which hydration shells replace bulk water, formed from combinations of different surfactants. These intermediate structures illustrate the continuous nature of the wRM‐to‐dRM transition rather than a discrete phase boundary, reflecting the gradual reduction of internal water content [22, 46]. This transition is directly reflected in RM dynamics and exchange behavior, as summarized in Figure 8: wRM exhibit rapid fusion–fission processes and dynamic exchange with the surrounding aqueous phase, whereas dRM display limited exchange and suppressed intermicellar dynamics due to reduced internal mobility.

**FIGURE 8:**
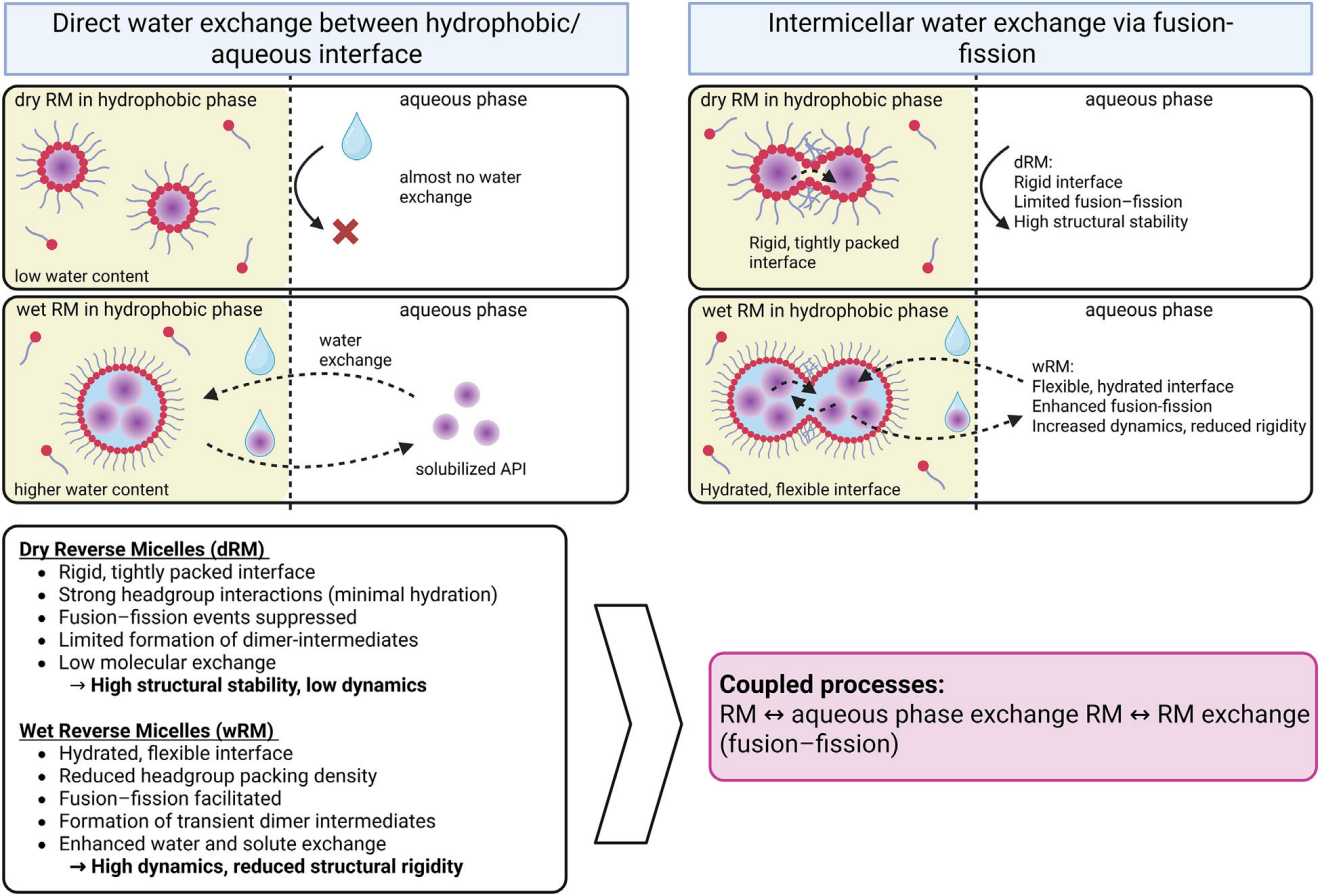
Hydration‐dependent exchange pathways in reverse micelles. The schematic contrasts direct water exchange between RM and the aqueous phase with intermicellar exchange via fusion–fission.

For pharmaceutical applications, formulations containing ≤ 1% (w/w) water are commonly classified as dRM within the investigated surfactant systems. Eczacioglu et al. [18] showed that this system‐specific threshold corresponds to a functional shift in RM behavior: above this level, RM retain a continuous aqueous domain with characteristic wRM dynamics, including rapid fusion–fission behavior and reversible rehydration, whereas below this level hydration shells replace bulk water and pronounced changes in stability, rehydration kinetics and interfacial dynamics emerge, defining the functional regime of dRM [18, 19, 22].

This shift from water‐dominated to interface‐dominated stabilization underlies the distinct structural and functional behavior of dRM compared to wRM. This operational definition provides a practical boundary for distinguishing hydration regimes in formulation development.

### 3.4 Thermodynamic Shift From wRM to dRM

The transition from wRM to dRM is accompanied by a profound change in the thermodynamic balance governing RM stability [44, 45, 77]. In wRM systems, stability is largely driven by the minimization of interfacial free energy between the internal aqueous phase and the surrounding hydrophobic phase, with water activity and entropy contributing significantly to RM size and dynamics [44, 45, 46, 77]. Increasing *w_0_* lowers interfacial curvature stress but increases internal entropy, resulting in a delicate balance that defines the stable RM size distribution [45, 77]. For example, the configurational entropy of the internal water pool promotes dynamic fusion—fission events, as reflected in the continuous coalescence—decoalescence behavior of RM and the composition‐dependent relaxation dynamics of confined water. Osmotically driven internal rearrangements further contribute to rapid structural fluctuations within the aqueous core [61, 68].

In dRM systems, this balance shifts markedly [46]. With diminishing water activity, entropic contributions from confined water become negligible, and stabilization is increasingly governed by direct interfacial interactions between surfactant headgroups and solutes [46, 84]. Typical examples include AOT–Na^+^ ion pairing, hydrogen‐bonded headgroup networks in phospholipid‐based systems and direct electrostatic or hydrophobic interactions between pP solutes and the surfactant interface, all of which provide enthalpic stabilization in the absence of bulk water. These enthalpic contributions replace the entropy‐driven stabilization characteristic of wRM [89, 90, 91].

## 4 Quality Attributes and Functional Advantages

The performance of RM relies on quality attributes (QAs) such as loading capacity, lipophilicity, protective effect, permeation enhancement and safety under gastrointestinal conditions [6, 17, 18, 34, 46], which together define whether a given RM meets the performance criteria summarized in Figure 9.

**FIGURE 9:**
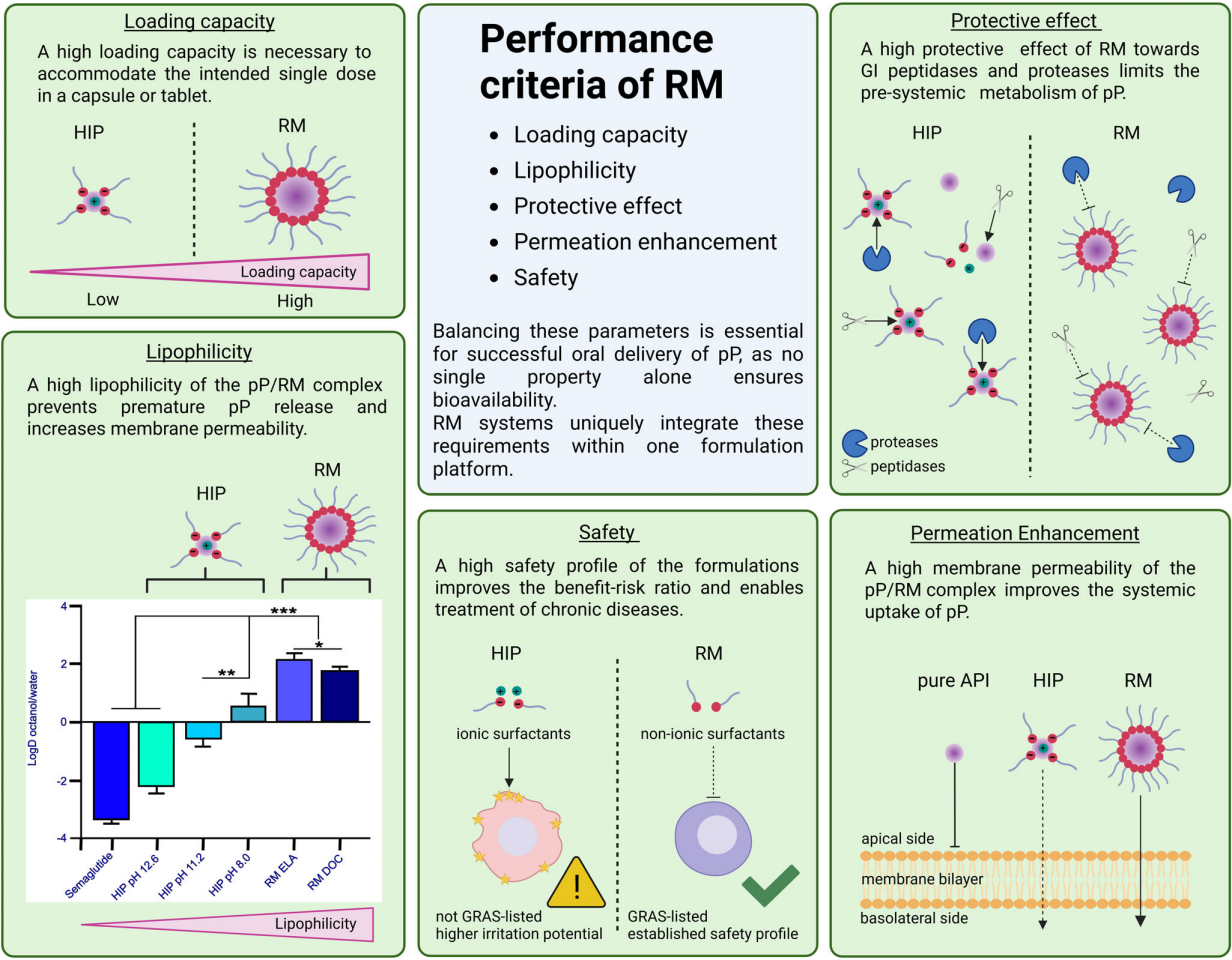
Performance criteria of RM relevant for oral delivery of pP therapeutics. RM performance is governed by five key quality attributes – loading capacity, lipophilicity, protective effect, permeation enhancement and safety – which together determine whether a RM formulation meets the functional requirements for successful GI‐transit and systemic uptake. Adapted from Sandmeier et al. [21].

### 4.1 Loading Capacity

Loading capacity is a key parameter for RM performance, defined as the maximum amount of pP that can be incorporated into the RM core under formulation‐relevant conditions. This becomes evident when examining dRM systems from Jörgensen et al. [17]: despite uniformly high initial entrapment efficiencies across broad surfactant concentrations, the maximum payload varies dramatically between surfactants and does not increase monotonically with surfactant content (Figure 10E). P85‐based dRM, for example, reach their highest payload at only 0.25% surfactant and then decline at higher concentrations, whereas AOT‐based dRM plateau around 5 %. In contrast, DODAB‐ and soy lecithin‐based dRM show a continuous increase in payload up to 10 %, reaching values several‐fold higher than in the pure hydrophobic phase. These differences arise from distinct solubilization mechanisms, ranging from hydrogen bonding in non‐ionic systems to combined ionic and interfacial interactions in charged surfactants.

**FIGURE 10:**
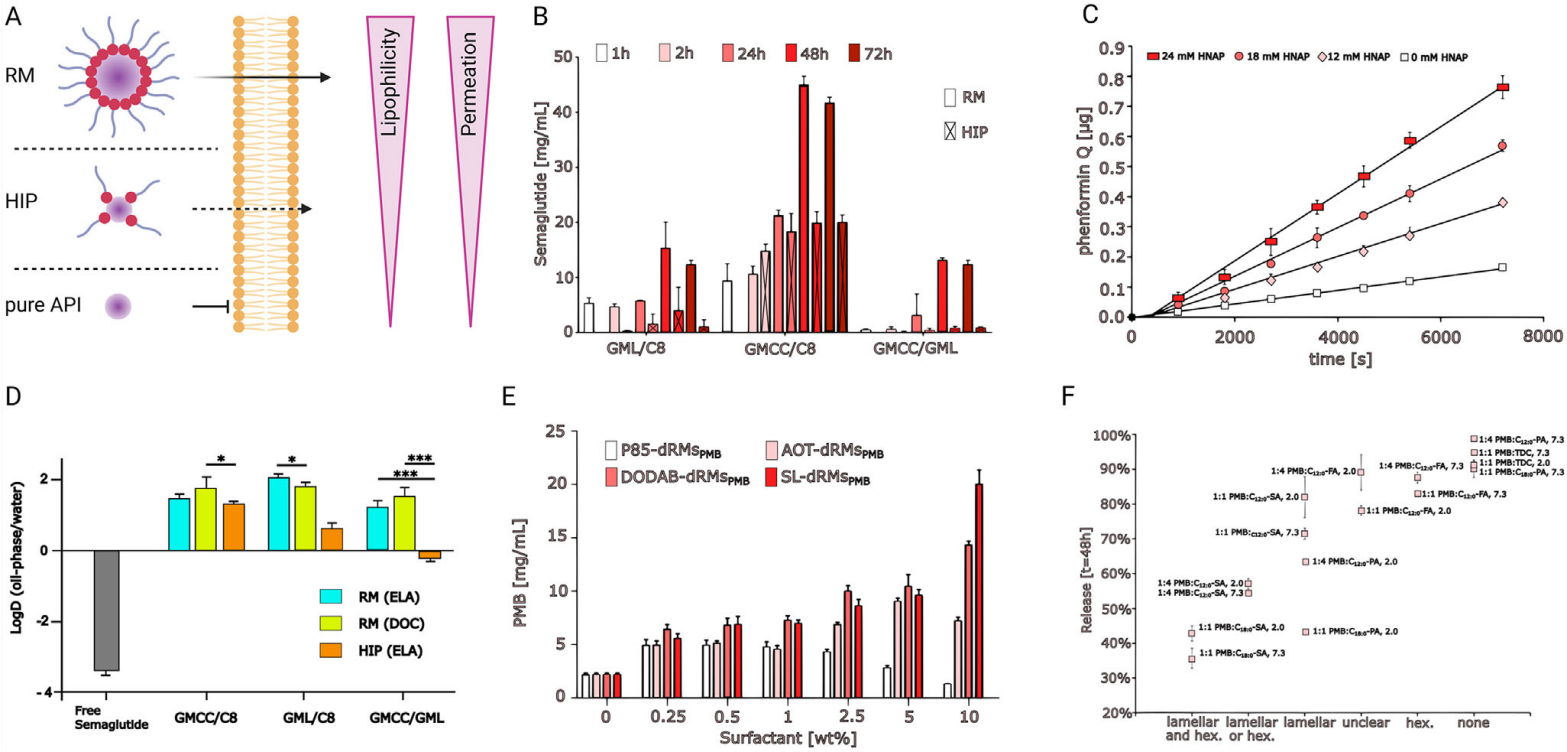
(A) Schematic illustration: RM markedly increase the apparent lipophilicity and membrane affinity of pP, enabling more efficient transcellular transport compared to HIP or free drug. (B) Solubility of a semaglutide within RM utilizing different incubation times. Adapted from Sandmeier et al. [21]. (C) Dependence of phenformin flux on counterion concentration in Caco‐2 cell assay; 24 mM HNAP (rectangle), 18 mM HNAP (circle), 12 mM HNAP (diamond) and 0 mM HNAP (square). Adapted from Miller et al. [92]. (D) Distribution coefficient logD_lipid phase/water_ of semaglutide as pure API, in RM (ELA and docusate) and HIP. Adapted from Sandmeier et al. [21]. (E) Maximum payloads of PMB in dRM formed at increasing surfactant concentrations. Adapted from Jörgensen et al. [17]. (F) Amount of PMB released after 48 h in each of the 16 release tests plotted against the liquid crystal phases present in the sample. Adapted from Ristroph et al. [93].

Beyond these structural effects, RM further enhance loading capacity by markedly increasing the apparent lipophilicity of pP. Sandmeier et al. [21] confirmed that RM increased pP lipophilicity almost twofold, resulting in a 4.2‐fold higher maximum payload in SEDDS compared to HIP (Figure 10B). This advantage arises from the dynamic interfacial solubilization environment of RM, characterized by adaptable curvature and hydration, whereas HIP systems are constrained by stoichiometric ion‐pair formation and therefore reach an intrinsic loading capacity (Figure 10A).

### 4.2 Lipophilicity

This enhanced loading capacity is mechanistically linked to the apparent logD, which governs pP partitioning during dilution and determines whether the maximum payload remains in the surrounding continuous phase or escapes into the aqueous phase. In RM, apparent logD is driven by interfacial affinity and surfactant headgroup chemistry: full interfacial embedding increases the effective lipophilicity of the pP, which directly enhances formulation stability and supports membrane interaction. In contrast, HIP increase lipophilicity solely through stoichiometric ion‐pair formation.

As shown by Miller et al. [92] for phenformin, increasing counterion concentration markedly raises apparent logD—by roughly 3.7 log units—and correspondingly increases Caco‐2 cell flux. However, both effects exhibit diminishing incremental gains at higher counterion concentrations, reflecting the stoichiometric limit of ion‐pair formation rather than a continuously tunable interfacial environment. This inherently caps the achievable lipophilicity, stability and membrane affinity of HIP systems compared to the dynamically organized RM (Figure 10C).

Sandmeier et al. [21] showed that RM increase pP lipophilicity by 3.5–5 log units relative to HIP‐systems, corresponding to a 3000‐ to 100 000‐fold higher distribution into the lipophilic phase (Figure 9 and Figure 10D).

Ebert et al. [19] further increased apparent logD within RM systems (Figure 10D), reporting values approaching 3 for RM‐formulated HRP, consistent with exceptionally strong membrane affinity. This elevated lipophilicity enables RM to interact more effectively with lipid bilayers and supports superior transcellular permeation compared to HIP.

### 4.3 Protective Effect

The protective effect of RM was confirmed by Groo et al. [24], who showed that pP encapsulated in RM lipid nanocapsules remained intact during trypsin exposure, whereas the free pP was rapidly degraded by proteases. Encapsulation also preserved the antimicrobial activity of AP138 against *Staphylococcus aureus*, including methicillin‐resistant strains (encapsulation efficiency 97.8% at a drug loading of 0.151%). This demonstrates that the RM environment effectively shields sensitive pP from proteolytic attack.

Ristroph et al. [93] demonstrated that formulations exhibiting lamellar or hexagonal liquid‐crystalline phases release PMB markedly more slowly, showing that increasing internal structural order progressively slows cargo release (Figure 10F). This structural rigidity protects pP from premature degradation by intestinal peptidases and proteases. Their liquid‐crystalline internal phase, with its highly ordered and viscous structure, is likely to enhance the overall protective effect. This supramolecular ordering will likely also reduce fusion–fission dynamics and inherently restrict enzymatic access to both the carrier surface and the encapsulated compounds.

Beyond these structural effects, the hydration state itself provides an additional layer of protection. HIP, where limited interfacial shielding leaves the payload more exposed, allow water to reach the API with greater ease, thereby facilitating hydrolytic degradation. In RM, tighter interfacial confinement and reduced water activity slow water penetration and provide substantially greater protection against premature chemical breakdown. In contrast to wRM, dRM contain no continuous aqueous phase. The absence of water markedly reduces hydrolytic degradation pathways, as both chemical amide‐bond hydrolysis and ester cleavage require a hydrated environment. Consequently, dRM can provide an intrinsically more stable microenvironment in which neither proteolytic enzymes nor water‐driven hydrolysis can readily occur.

### 4.4 Permeation Enhancement

Permeation of pP across intestinal membranes correlates strongly with apparent logD, as octanol–water partitioning reflects affinity for the intestinal phospholipid layer. RM formulations achieve substantially higher apparent logD values than HIP, providing a stronger thermodynamic drive for membrane interaction. This increase in apparent logD arises from interfacial embedding rather than stoichiometric ion‐pair formation. Miller et al. [92] demonstrated that increasing lipophilicity via ion pairing enhances membrane permeation; however, in RM systems this effect is achieved through interfacial embedding rather than stoichiometric complexation. As a result, RM enable substantially higher and more tunable apparent logD values, which promote stronger association with phospholipid bilayers and facilitate transcellular transport.

In lipid‐based formulations such as SEDDS, this effect is further amplified by the high fraction of lipophilic surfactants and lipid surroundings, which favor direct interaction with cellular membranes. Upon dispersion in gastrointestinal fluids, RM‐containing droplets can closely approach and partially merge with the intestinal epithelium, enabling transfer of the encapsulated payload across the membrane. Their high interfacial fluidity and lipophilic composition facilitate partial penetration into the mucus layer, which functions as an amphiphilic gel network and is inherently more permeable to lipid‐rich colloids than to rigid hydrophilic or particulate carriers [36]. Experimental evaluations by Griesser et al. explicitly demonstrate that fluid lipid droplets exhibit significantly higher mucosal permeation rates compared to rigid polymeric nanoparticles or liposomes [94]. This enables RM‐containing droplets to diffuse through the mucus barrier without requiring active mucolytic disruption and to reach the apical epithelial surface. There, their surfactant‐rich interfaces generate localized curvature stress and promote transient lipid mixing with the enterocyte membrane [95].

In contrast to particulate delivery systems that rely primarily on endocytosis, some RM‐containing lipid systems may promote direct membrane interaction through interfacial mixing or other formulation‐dependent processes. However, the dominant transport mechanism cannot be generalized across all RM‐based architectures. This provides a possible explanation for enhanced membrane interaction in selected RM‐containing formulations, although the contribution of the RM component cannot be separated from that of the surrounding lipid and surfactant matrix in complex systems. These findings can be considered in the context of the study by Lu et al. [26], who developed a double‐layer nanoparticle system comprising RM‐loaded lipid cores surrounded by an outer hydrophilic shell. The observed cellular uptake and in vivo efficacy likely reflect the combined effects of the RM‐containing lipid core, the nanoparticle architecture, and the hyaluronic acid coating. Although RM may contribute to payload incorporation and interfacial organization, these outcomes cannot be attributed to RM alone (Figure 10H,I). The hyaluronic acid coating further enhances this effect by improving mucus interaction and facilitating receptor‐mediated uptake, thereby increasing local residence time at the epithelial surface and promoting internalization [26].

Importantly, this suggests that RM do not merely act as passive solubilization compartments but actively contribute to cellular uptake processes. By increasing apparent lipophilicity, stabilizing the payload within a membrane‐compatible microenvironment and enabling dynamic interfacial interactions, RM‐based systems provide a dual advantage: efficient retention in the hydrophobic phase combined with enhanced transfer across biological barriers. This combination distinguishes RM from conventional strategies and highlights their potential to overcome one of the central limitations in oral delivery of pP.

### 4.5 Safety

These formulation advantages are further supported by the favorable safety and biocompatibility profile of the surfactants used in dispersed RM. Surfactants commonly employed in RM systems for oral lipid‐based formulations are typically GRAS‐listed, exhibit low intrinsic toxicity and are well characterized in terms of their metabolic fate. This may provide a regulatory advantage over approaches relying on less well‐characterized excipients. However, not all surfactants used for RM formulation are GRAS‐listed or have an established history of oral use; therefore, the safety profile of each RM formulation must be evaluated on the basis of its specific excipient composition. Moreover, GRAS status of individual excipients does not establish the long‐term safety of the corresponding pP‐loaded RM formulation, and dedicated repeated‐dose and chronic exposure studies remain limited. Comparative studies show that dRM formulations can outperform wRM in maintaining stability and biological activity, supporting their suitability for sensitive oral payloads [15, 18, 22].

The relevance of excipient safety becomes even more pronounced when considering chronic indications, where daily, lifelong administration imposes far stricter requirements than acute or short‐term therapies. Although established safety profiles of individual excipients may facilitate formulation development, their safety cannot be assumed to remain unchanged following incorporation into RM or during prolonged exposure. For RM formulations containing surfactants without an established GRAS status or history of oral administration, dedicated toxicological characterization is therefore particularly important before clinical translation. In contrast, HIP may rely on counterions lacking GRAS status, increasing the need for dedicated toxicological characterization and potentially adding regulatory complexity, particularly for repeated‐dose or lifelong therapies.

## 5 In Vivo Performance of RM‐Based Systems

The available in vivo evidence includes both classical RM formulations and more complex RM‐based delivery systems, such as self‐emulsifying lipid phases, nanocapsules, and surface‐modified nanoparticles. In the latter systems, the observed performance reflects the combined effects of RM incorporation and the surrounding formulation components, including the lipid matrix, emulsification properties, and additional surface functionalities. Therefore, these findings should not be interpreted as evidence for the efficacy of RM alone. The potential of RM for the oral delivery of hydrophilic pP has increasingly been investigated in vivo. While early studies primarily focused on encapsulation, stabilization, and membrane interaction, recent work has demonstrated that these formulation effects can translate into measurable pharmacokinetic and pharmacodynamic responses following oral administration. Importantly, the available evidence also indicates that in vivo performance is strongly dependent on formulation architecture, including surfactant chemistry, internal water content, lipid composition, and self‐emulsification behavior. Direct comparisons with HIP, as well as comparisons between dRM and wRM, therefore provide an important basis for critically evaluating the potential and limitations of RM‐based systems for oral delivery [21, 28].

### 5.1 RM versus Hydrophobic Ion Pairs

HIP is an established strategy for increasing the lipophilicity of hydrophilic pP and facilitating their incorporation into lipid‐based drug delivery systems. In HIP, ionizable groups of the pP are associated with oppositely charged hydrophobic counterions, thereby increasing the apparent lipophilicity of the resulting complex. The extent of this effect is therefore strongly influenced by the charge state of the pP and the chemical properties and stoichiometry of the counterion [96, 97].

RM provide a different formulation architecture in which the hydrophilic pP is associated with the organized polar interface of the surfactant assembly. Importantly, this distinction does not imply that electrostatic interactions are absent from RM. Charged surfactants may interact electrostatically with oppositely charged regions of the pP and thereby contribute to its stabilization within the RM [6, 17, 21]. However, unlike classical HIP, RM incorporation does not require the formation of a defined stoichiometric peptide–counterion complex. Instead, electrostatic interactions can act alongside hydrogen bonding, hydration, and other non‐covalent interactions within the surfactant interface [6, 17]. Thus, RM can exploit ionic interactions without being restricted to a charge‐dependent ion‐pairing mechanism. This distinction is particularly relevant for pP containing multiple charged and polar regions, for which interfacial association may involve several interaction sites rather than a single defined ion pair [17, 21].

The practical consequences of these different architectures were directly demonstrated by Sandmeier et al. [21], who compared RM and HIP for the incorporation of semaglutide into SEDDS. RM were prepared using either the cationic surfactant ELA or the anionic surfactant AOT, whereas HIP were formed between semaglutide and ELA. RM achieved substantially higher peptide payloads than HIP, with maximum values of 24.2 ± 8.8 mg mL^−1^ for ELA‐based RM and 16.3 ± 6.0 mg mL^−1^ for DOC‐based RM, compared with 6.3 ± 0.2 mg mL^−1^ for the HIP formulation. RM also provided an approximately twofold higher apparent lipophilicity and resulted in a 4.2‐fold higher SEDDS payload compared with HIP. Importantly, the study demonstrated that the charge of the surfactant still influences RM incorporation, as the cationic ELA‐based RM exhibited higher lipophilicity than the anionic DOC‐based system. Thus, the findings provide a direct example of electrostatic contributions to RM stabilization while simultaneously demonstrating that RM incorporation is not equivalent to classical HIP.

The broader literature further illustrates the formulation‐dependent nature of RM incorporation (Table 2). Jörgensen et al. [17] investigated polymyxin B (PMB) in dRM prepared with non‐ionic P85, anionic AOT, cationic DODAB and soy lecithin. Although high entrapment efficiencies were observed across the investigated systems, maximum payloads differed substantially between surfactants, indicating that RM loading is strongly dependent on interfacial chemistry. In particular, the authors associated the observed differences with distinct solubilization mechanisms, ranging from hydrogen bonding in non‐ionic systems to combined ionic and interfacial interactions in charged surfactants. These findings further support the concept that electrostatic interactions can contribute to RM stabilization without defining the entire incorporation mechanism.

**TABLE 2:** Comparative Performance of RM and HIP for pP Delivery.

Study	Payload/model	Strategy	Surfactant/counterion	Maximum payload	Lipophilicity	Cytocompatibility/safety	In vivo performance	Main finding/limitation
Jörgensen et al. [17]	Polymyxin B (PMB)	dRM	P85, AOT, DODAB, soy lecithin	20.01 ± 1.35 mg mL^−1^ (SL, 10%); 14.33 ± 0.37 mg mL^−1^ (DODAB, 10%); 9.02 ± 0.31 mg mL^−1^ (AOT, 5%); 4.91 ± 0.56 mg mL^−1^ (P85, 0.25%)	Increased vs free PMB; exact values depend on surfactant	AOT caused >50% viability loss ≥0.1%; P85/SL complete cell death at 0.5%; DODAB ∼50% viability at 0.5%	Not reported	dRM payload reached ∼7‐fold the HIP benchmark, but performance strongly depended on surfactant and concentration.
Eczacıoğlu et al. [18]	HRP + model dyes	dRM vs wRM	Cationic, amphoteric and non‐ionic surfactants; HRP system with Span 80	SEDDS‐dRM: 11.2%; SEDDS‐wRM: 7.9%	dRM model system: MB logD 1.56 vs wRM 0.59	>90% cell survival for SEDDS‐dRM up to 0.5%; wRM caused complete cell death >0.4%	HRP oral F_a_: 11.2% dRM, 7.9% wRM	dRM showed higher HRP bioavailability despite lower apparent permeability in the model system; demonstrates that water state strongly affects performance.
Sandmeier et al. [21]	Semaglutide	RM vs HIP	RM: ELA or DOC; HIP: ELA	RM: 24.2 ± 8.8 mg mL^−1^ (ELA), 16.3 ± 6.0 mg mL^−1^ (AOT); HIP: 6.3 ± 0.2 mg mL^−1^	RM approximately 2‐fold higher lipophilicity than HIP; HIP logD around 1 in the SEDDS comparison	>75% Caco‐2 survival up to 0.1%; acceptable hemolysis for both	Not reported	Direct evidence that RM can exploit charge‐dependent interactions while achieving substantially greater payload/lipophilicity than HIP.
Ricci et al. [22]	HRP	mixed dRM	Soybean Phosphati‐ dylcholine+ AOT	3% w/v HRP in lipophilic phase	HRP logD increased from −3.36 to 3.10	Not reported	Not reported	Demonstrates exceptionally large lipophilicity shift and high protein retention without organic cosolvent.
Groo et al. [24]	AP138	RM lipid nanocapsules	AOT‐based RM	Drug loading 0.151%; EE 97.8%	Not reported	Not the principal endpoint	Not directly comparable to oral PK table	Strong evidence for proteolytic protection, but relatively low mass loading.
Lu et al. [26]	Exe	RM‐SEDDS/ RM‐O/W‐HA	Soy phosphor lipid; RH40/ Labrasol; DOTAP; HA	Not reported	Not reported	Reported good safety; exact comparative viability values not central	Significant glucose‐lowering effect in T2DM rats; RM‐O/W‐HA stronger/ prolonged response	Biological activity and uptake, but formulation is structurally more complex than a conventional RM‐SEDDS.
Lindner et al. [27]	Hydrophilic model drugs	dRM	Multiple surfactant systems	Solvent‐assisted preparation achieved up to 109‐fold higher solubilization than dry addition	Not the principal endpoint	Not reported	Not reported	Preparation route itself is a major determinant of loading, limiting simple cross‐study comparisons.
Schmidt et al. [28]	Exe	dRM vs HIP in SEDDS	dRM: Span 80; HIP: cationic counterion	Fa: dRM 17.06%, HIP 18.08%; payload values formulation‐dependent	logDSEDDS/AQ: 2.05 ± 0.08 dRM vs 2.13 ± 0.31 HIP	Low toxicity in tested systems	Relative F_a_: 17.06% dRM, 18.08% HIP	Counterexample: HIP slightly exceeded dRM in bioavailability, demonstrating that RM not universally superior.
He et al. [29]	Exe	RM‐LNC / Folic‐acid‐RM‐LNC	Soy phospholipid‐based RM	Not reported	Not reported	Good safety reported	RM‐LNC increased exposure; FA‐RM‐LNC further increased exposure and prolonged plasma levels; exact values in your review are figure‐derived estimates	Shows that RM architecture can be combined with targeting/mucoadhesive strategies, but direct comparison with simple RM is difficult.
Wang et al. [30]	Insulin	anhydrous/ phospholipid RM	Phosphatidylcholine	Not reported	Not reported	Not reported	Oral dRM reduced blood glucose to ∼71.3% of baseline at 4 h, with effect up to 12 h	Demonstrates preserved biological activity but provides no PK/bioavailability data.
Miller et al. [92]	Phenformin	HIP	1‐hydroxy‐2‐naphthoic acid	Not applicable	+3.7 log units in lipophilicity with HNAP	Not the focus	Not reported	Useful mechanistic HIP benchmark, but not a peptide/protein study, so should not be directly equated with RM peptide studies.

Similarly, Ricci et al. [22] demonstrated that mixed dRM containing HRP, soybean phosphatidylcholine, and sodium docusate can markedly alter the apparent lipophilicity of a highly hydrophilic protein. Incorporation shifted the distribution coefficient of HRP from logD = −3.36 to 3.10, corresponding to a calculated increase of 6.46 log units, while more than 95% of the incorporated protein remained intact following proteolytic challenge. These findings illustrate that RM can simultaneously provide a lipophilic environment and protect structurally sensitive biomolecules, although the study did not include a direct HIP comparator.

Additional evidence for the potential of RM‐based architectures comes from He et al. [29], who investigated folic acid‐modified RM lipid nanocapsules for oral delivery of Exe. Both unmodified RM‐LNC and folic acid‐modified RM‐LNC increased systemic exposure compared with orally administered Exe solution, with the folic acid‐modified formulation producing the highest plasma concentrations over 24 h (Figure 11B). These findings support the ability of RM‐based nanocarriers to combine gastrointestinal protection with enhanced epithelial uptake; however, because no direct HIP comparator was included, they do not establish superiority over ion‐pair‐based systems.

**FIGURE 11:**
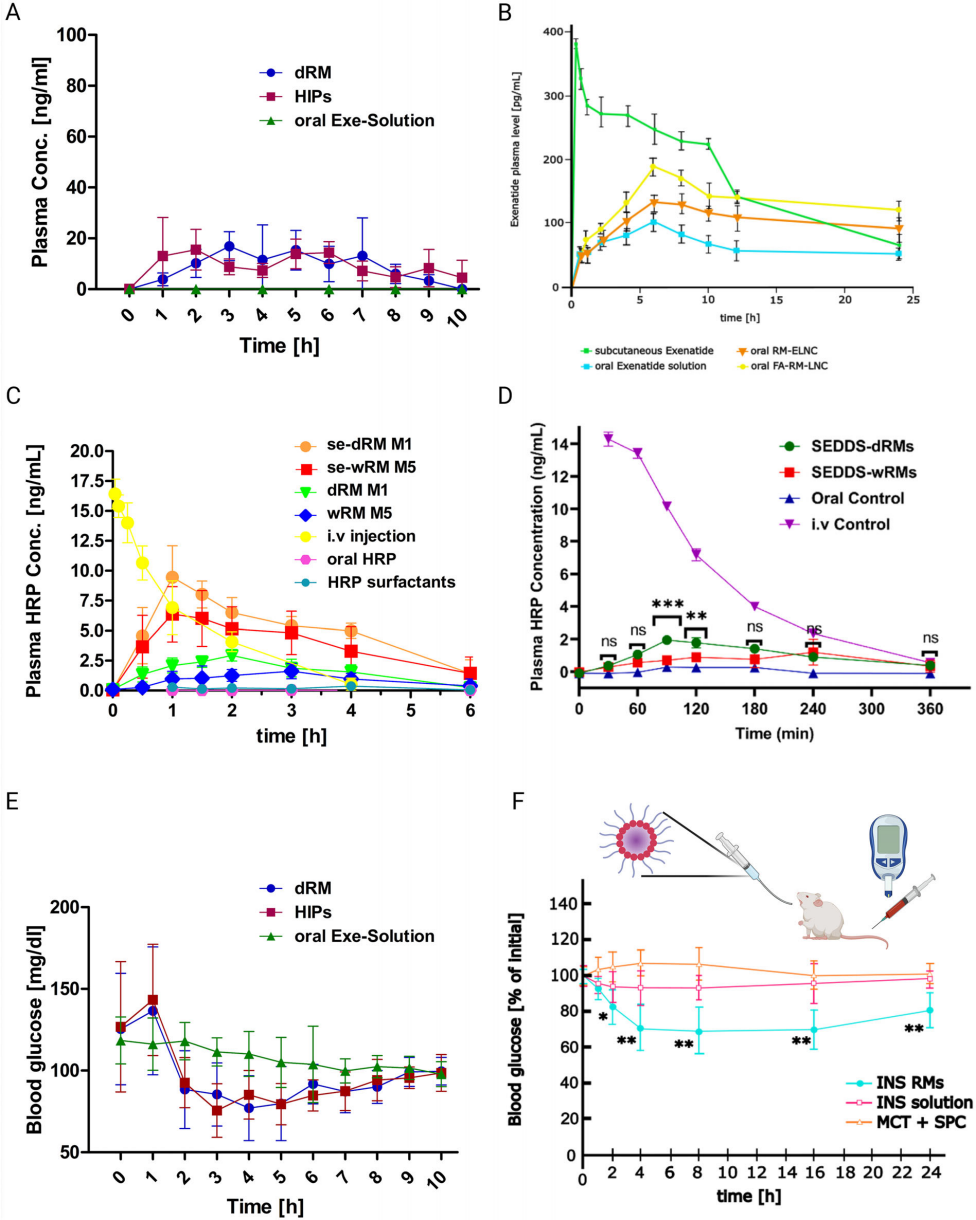
(A) Exe plasma concentration–time curve for orally administered Exe‐SEDDS formulations with HIP (red) and RM (blue) as well as aqueous Exe solution (green). Reproduced from Schmidt et al. [28] under the terms of the Creative Commons CC BY license. (B) Plasma Exe concentration–time profiles following subcutaneous (SC) and oral administration of Exe formulations in rats. Adapted from He et al. [29]. (C) In vivo performance of HRP‐loaded RM and reference solutions. Plasma concentration–time profiles of horseradish peroxidase (HRP) following intravenous injection of sterile HRP solution and oral administration of HRP solution. Reproduced from Ebert et al. [19] under the terms of the Creative Commons CC BY license. (D) HRP blood profile measured using QuantaRed kit over time after oral administration to rats in solution in water (Oral Control) or encapsulation within SEDDS‐dRM and SEDDS‐wRM. Reproduced from Eczacioglu et al. [18] under the terms of the Creative Commons CC BY license. (E) Blood glucose level after oral administration of Exe‐SEDDS formulations with HIP (red) and dRM (blue) as well as aqueous Exe solution (green). Reproduced from Schmidt et al. [28] under the terms of the Creative Commons CC BY license. (F) Changes in blood glucose level versus time profiles following oral administration of insulin‐containing dRM in diabetic male Wistar rats (*n*=5) fasted for 24 h. Adapted from Wang et al. [30].

In contrast, a direct comparison by Schmidt et al. [28] illustrates that the advantages of RM are not universal. Exe‐loaded dRM and HIP were incorporated into the same SEDDS platform and exhibited very similar maximum apparent logD values of 2.05 ± 0.08 and 2.13 ± 0.31, respectively. Following oral administration (Figure 11A), relative bioavailability was likewise comparable, reaching 17.06% for dRM and 18.08% for HIP. Thus, despite their fundamentally different incorporation mechanisms, both approaches achieved a similar level of systemic exposure. HIP additionally provided more pronounced control over Exe release, whereas the dRM formulation was based on sorbitan monooleate and other excipients selected for favorable safety and compatibility, while both strategies effectively reduced blood glucose levels (Figure 11E) through glucose‐dependent insulin secretion and demonstrated strong potential as protective oral nanocarriers for type 2 diabetes therapy.

Taken together, these studies indicate that RM should not be regarded as universally superior to HIP, but rather as a complementary strategy with distinct formulation advantages. RM can achieve high payloads and substantially modify the apparent lipophilicity of hydrophilic biomolecules, as demonstrated for semaglutide, PMB, and HRP [17, 21, 22]. At the same time, direct comparison with Exe demonstrates that improved loading or an alternative incorporation mechanism does not necessarily translate into higher oral bioavailability [28]. The relative performance of RM and HIP therefore depends on the physicochemical properties of the pP, surfactant and counterion chemistry, lipid composition, preparation method, and the biological endpoint considered. This formulation dependence should be taken into account when evaluating RM as an alternative to established ion‐pairing approaches.

### 5.2 Dry versus Wet RM

A particularly relevant question is whether reducing the internal water content of RM from a hydrated to a near‐anhydrous state translates into improved oral performance. This distinction is important because water content does not merely define the physical state of the RM; it also influences surfactant organization, molecular mobility, payload retention, and the behavior of the system upon exposure to gastrointestinal fluids. Recent studies directly comparing dry and wet RM provide increasing evidence that reduced hydration can favor the stability and functional performance of RM‐based lipid formulations. Table 3 provides an overview of the corresponding in vivo pharmacokinetic parameters and formulation characteristics evaluated across these studies.

**TABLE 3:** Pharmacokinetic parameters of RM‐based in vivo studies.

Formulation	Payload	BA	c_max_	T_max_	Refs.
SEDDS containing dRM; RM formed with Span 80 in lipid phases isocetyl stearate or MCT/MCM, incorporated into SEDDS lipid matrix	HRP	11.20% F_a_	1.90 ng mL^−1^ (estimated)	2 h	[18]
SEDDS containing wRM; same surfactant system (Span 80) but with water uptake forming wet RM in isocetyl stearate or MCT/MCM lipid phase, incorporated into SEDDS	HRP	7.90% F_a_	1.00–1.20 ng mL^−1^ (estimated)	2‐4 h	[18]
SEDDS containing dRM; lipid matrix propylene glycol dilaurate + salicylic acid methyl ester (32.5:32.5) with polyethoxylated‐35 castor oil (35%) as surfactant	Exe	17.06% F_a_	16.91 ± 8.00 ng mL^−1^	3 h	[28]
SEDDS containing HIP; same SEDDS matrix (propylene glycol dilaurate + salicylic acid methyl ester + Cremophor EL) with tetraoctylammonium bromide counterion	Exe	18.08% F_a_	15.58 ± 5.75 ng mL^−1^	2 h	[28]
dRM with Span80 in IPM	HRP	1.54 ± 0.22% F_a_	2.95 ± 0.48 ng mL^−1^	2 h	[19]
wRM with Span80 in IPM/MCT mixture (20:80)	HRP	0.89 ± 0.20% F_a_	1.62 ± 0.34 ng mL^−1^	3 h	[19]
dRM in self‐emulsifying system (se‐dRM M1); same dry RM system plus self‐emulsifying surfactant matrix	HRP	12.10 ± 1.94% F_a_	9.46 ± 1.64 ng mL^−1^	1 h	[19]
wRM in self‐emulsifying inverted lipid phase (se‐wRM M5); wet RM	HRP	9.24 ± 3.82% F_a_	6.37 ± 2.31 ng mL^−1^	1 h	[19]
RM‐LNC (oral)	Exe	↑ vs. Exe solution (∼2%–3%^*^) F_r_	Low; increased vs. Exe solution	∼2 h^*^	[29]
FA‐RM‐LNC (oral, PEG‐FA modified)	Exe	↑↑ (∼4%–6%^*^) F_r_	Higher than RM‐LNC (∼1.5–2×^*^)	∼3–4 h^*^	[29]
RM‐SEDDS (reverse micelle–based self‐emulsifying system; surfactant‐stabilized RM embedded in lipid phase)	Exe	Not reported	Not reported		[26]
HA‐coated RM‐O/W nanoparticles (double‐layer system: RM‐loaded oil droplets with outer hyaluronic acid shell for mucus interaction and targeting)	Exe	Not reported	Not reported		[26]
Phospholipid‐based ARM from lyophilized W/O emulsions (oral lipid formulation, SPC/MCT system) Oral administration (diabetic rat model); significant reduction in blood glucose levels observed. Maximum pharmacodynamic effect (71.3% of baseline) at 4 h, with sustained activity up to 12 h. Corresponds to a PD T_max_ ≈ 4 h (no PK T_max_ reported)	Insulin	Not reported	Not reported	Not reported	[30]

* estimated from Figure 11B (plasma concentration‐time curve); relative bioavailability (against s.c. injection).

Ebert et al. [19], recently investigated the influence of hydration state on the oral performance of RM‐based inverted lipid phases using HRP as a model pP (Figure 11C). The study compared dry and wet RM‐containing lipid phases as well as corresponding self‐emulsifying formulations. While the non‐self‐emulsifying systems exhibited limited emulsification and low oral bioavailability, incorporation of an additional non‐ionic emulsifier markedly improved gastrointestinal dispersion and systemic exposure.

Within these self‐emulsifying systems, the dry formulation achieved a relative oral bioavailability of 12.10%, compared with 9.24% for the corresponding wet formulation, demonstrating that reduced hydration can translate into a measurable pharmacokinetic advantage in vivo. Importantly, the study also showed that efficient gastrointestinal self‐emulsification is a prerequisite for this benefit, highlighting that the hydration state of the RM cannot be considered independently of the emulsification behavior of the overall lipid carrier.

Direct evidence for the functional consequences of the dry‐to‐wet transition was provided by Eczacıoğlu et al. [18], who systematically compared dRM and wRM within SEDDS using several physicochemical and biological endpoints (Figure 11D). Sorbitan monooleate‐based dRM exhibited a lower water content and substantially reduced water uptake compared with wRM, accompanied by higher apparent lipophilicity and entrapment efficiency. Upon incorporation into SEDDS, these differences were reflected in improved cytocompatibility and, importantly, in vivo performance. Oral administration of HRP‐loaded SEDDS resulted in a relative bioavailability of 11.2% for dRM compared with 7.9% for wRM, corresponding to a 1.42‐fold higher systemic exposure for the dry formulation. Although the study does not establish a single molecular mechanism responsible for this difference, the superior performance of dRM is consistent with their greater payload retention and reduced structural reorganization upon exposure to aqueous and gastrointestinal conditions [18].

This distinction is particularly relevant because the two RM states exhibit markedly different hydration‐dependent physicochemical behavior. The higher water uptake and larger internal aqueous domain of wRM facilitate dynamic structural rearrangement and molecular exchange, whereas the reduced hydration of dRM favors a more interfacially confined state. The resulting differences in payload localization, apparent lipophilicity, and retention may therefore influence the fate of RM during gastrointestinal dispersion and digestion. Notably, the Eczacıoğlu study found that wRM exhibited higher membrane permeability than dRM in the Transwell model despite their lower oral bioavailability, illustrating that enhanced permeability alone does not determine systemic exposure. Rather, successful oral delivery depends on the combined effects of payload retention, protection, gastrointestinal stability, emulsification, and epithelial transport [18].

The in vivo relevance of RM‐based peptide delivery is further supported by studies that did not directly compare dry and wet systems. Lu et al. [26] developed Exe‐loaded phospholipid RM incorporated into SEDDS and subsequently into HA‐coated double‐layer nanoparticles. The RM‐based systems produced a pronounced and sustained glucose‐lowering response in type 2 diabetic rats, while the HA‐coated formulation further enhanced cellular uptake through CD44‐mediated endocytosis. Although this study does not provide a direct dRM–wRM comparison, it demonstrates that RM‐based architectures can preserve the biological activity of an orally administered pP and translate RM incorporation into a measurable pharmacodynamic effect [26].

An earlier study by Wang et al. [30], likewise demonstrated the potential of anhydrous reverse micelles (ARM) for oral peptide delivery. Insulin‐loaded ARM were prepared by lyophilization of water‐in‐oil emulsions containing phosphatidylcholine. Following oral administration to diabetic rats, the formulation reduced blood glucose to approximately 71.3% of baseline within 4 h, with the effect maintained for up to 12 h, whereas insulin solution produced only a limited and shorter‐lived response (Figure 11F). Although pharmacokinetic parameters were not determined and no wet RM comparator was included, these findings provided early evidence that removal of water from a phospholipid‐based RM system can yield an oil‐compatible formulation capable of preserving the biological activity of a peptide following oral administration [30].

Taken together, the available evidence supports a functional distinction between wet and dry RM rather than a simple binary classification based on water content. Direct comparisons demonstrate that dRM can provide higher apparent lipophilicity, improved payload retention and, in SEDDS, higher oral bioavailability than wRM. However, the magnitude of this advantage depends strongly on the surrounding lipid formulation and its ability to self‐emulsify under gastrointestinal conditions. Thus, dehydration should not be considered an intrinsically beneficial formulation step in isolation; rather, the transition from wRM to dRM appears advantageous when the resulting interfacial organization improves payload retention while maintaining efficient dispersion and release after oral administration.

### 5.3 Limitations and Translational Challenges

Despite the promising performance of RM‐based systems, their successful application is not universal and remains highly dependent on formulation design. Importantly, RM formation and payload retention do not necessarily translate into efficient oral delivery. Ebert et al. [19] demonstrated that the oral performance of RM‐containing lipid phases was strongly influenced by the surrounding lipid formulation and its self‐emulsification capacity. Formulations with insufficient emulsification capacity exhibited limited gastrointestinal dispersion and low oral bioavailability, whereas incorporation of an additional non‐ionic emulsifier improved systemic exposure. These findings indicate that the observed in vivo performance results from the combined effects of RM incorporation, lipid composition, and self‐emulsification behavior, rather than from RM alone.

Moreover, RM do not consistently outperform established approaches such as HIP. In a direct comparison using Exe‐loaded SEDDS, Schmidt et al. [28] reported comparable relative oral bioavailability for dRM (17.06%) and HIP (18.08%), while HIP provided more pronounced control over peptide release. Thus, RM should be considered a complementary rather than universally superior strategy, with the relative performance depending on peptide properties, surfactant chemistry, formulation composition, and the intended therapeutic endpoint.

Translation to scalable manufacturing also presents challenges. The incorporation of hydrophilic pP into RM can be strongly dependent on the preparation method, with conventional dry addition requiring prolonged equilibration, whereas solvent‐assisted approaches can substantially accelerate loading but introduce additional processing steps related to solvent compatibility and removal [21, 27]. Such differences may affect process time, batch‐to‐batch reproducibility, and manufacturing control at larger scales. Consequently, preparation conditions that are effective at laboratory scale cannot necessarily be assumed to translate directly to robust and scalable manufacturing processes.

An additional consideration for translation is the robustness of RM‐containing self‐emulsifying systems during processing and storage. Although SEDDS are established lipid‐based delivery systems with substantial pharmaceutical development experience, their physicochemical stability and handling can remain challenging during storage and formulation development [98, 99]. The incorporation of pP‐loaded RM introduces an additional level of interfacial organization that may be sensitive to formulation composition and processing conditions, as reflected by the pronounced influence of surfactant chemistry on the properties of RM‐containing SEDDS [17]. Changes in water content, temperature, mixing conditions, or excipient composition could potentially affect RM organization, payload retention, and subsequent self‐emulsification behavior. However, systematic studies addressing the processing robustness, long‐term storage stability, and scale‐up of RM‐containing SEDDS remain scarce. Accordingly, these aspects represent important translational considerations rather than established limitations of RM technology.

Finally, the current evidence base remains predominantly preclinical and is derived from a relatively limited number of model pP and animal studies [18, 19, 26, 28, 30]. Long‐term stability, repeated‐dose safety, large‐animal pharmacokinetics, and ultimately clinical performance remain insufficiently investigated. Accordingly, RM should currently be regarded as a promising but still largely preclinical platform, for which systematic optimization of formulation robustness, manufacturability, and biological performance will be essential for successful translation.

## 6 Future Directions

The most suitable mucosal delivery systems for RM are likely lipid‐based nanocarriers. Their small size facilitates permeation through the mucus gel layer, which has a characteristic mesh size of approximately 100–500 nm [100]. Relevant carrier systems include SEDDS, solid lipid nanoparticles (SLN), nanostructured lipid carriers (NLC), and lipid‐based nanoparticles (LNPs).

Upon reaching the absorption membrane, these nanocarriers can be internalized by epithelial cells via endocytosis. Following uptake, they may undergo transcytosis –transport across the cell followed by exocytosis at the basolateral side—or escape from endosomes into the cytoplasm, enabling their cargo to reach systemic circulation via permeation across the basolateral membrane. However, both transcytosis and endosomal escape are generally inefficient processes and therefore limit overall systemic uptake.

An alternative pathway involves direct fusion of the nanocarrier with the cellular membrane, enabling immediate release of the payload into the cytoplasm while bypassing endosomal trafficking. Efficient membrane fusion requires high carrier fluidity, as observed in systems such as SEDDS or oil‐in‐water (O/W) emulsions. In addition, a cationic surface charge—introduced via cationic surfactants—can enhance interactions with negatively charged cell membranes. Surfactants with an inverted cone geometry further promote negative membrane curvature, which is favorable for fusion [101]. A fundamental challenge arises, however: the formation of highly fluid, lipid‐based droplets typically relies on cone‐shaped surfactants that induce positive curvature, opposing the negative curvature associated with early membrane‐fusion intermediates such as the stalk. RM may help resolve this apparent contradiction. Their formation is governed by inverted cone‐shaped surfactants that favor negative spontaneous curvature, while maintaining a lipidic interior. This curvature preference could, in principle, facilitate interactions with cellular membranes and lower the energetic barrier for membrane‐fusion intermediates. However, membrane fusion is governed by a complex interplay of lipid composition, spontaneous curvature, membrane hydration, and interfacial interactions, and the ability of RM to directly fuse with intestinal membranes therefore remains a hypothesis requiring further experimental validation. Supporting this hypothesis, a study specifically investigating membrane‐fusion RM/SEDDS reported evidence for membrane‐associated internalization of hydrophilic peptides and examined cellular uptake in the presence of endocytosis inhibitors [102]. These findings provide a basis for considering membrane fusion as a possible contribution to RM‐mediated cellular uptake, rather than establishing it as the sole mechanism. Nevertheless, to fully exploit this approach, formulations must be further optimized and tailored to specific applications.

In addition, many permeation enhancers used for therapeutic pP—such as medium‐chain fatty acids, bile salts, sodium dodecyl sulfate, or SNAC [103]—are surfactants that can be readily incorporated into RM. This creates the potential for synergistic effects. Importantly, incorporation into RM enables co‐delivery of both the therapeutic molecule and the permeation enhancer to the absorption site. This is particularly advantageous, as permeation enhancers are otherwise rapidly diluted in gastrointestinal fluids and often absorbed more quickly than the pP drug. Such synchronized delivery may therefore substantially improve absorption efficiency.

Beyond pP, RM may also be applied to other hydrophilic macromolecules. In particular, oral or nasal delivery of nucleic acids (e.g., mRNA, aptamers) and polysaccharides such as low‐molecular‐weight heparin could significantly improve patient compliance compared with injectable formulations. Notably, currently used lipid nanoparticles for mRNA delivery—such as those employed in COVID‐19 vaccines—contain inverted lipid phases that share structural similarities with RM [104]. Thus, the design principles established for reverse micelle‐based delivery of pP may serve as a blueprint for the development of non‐invasive delivery systems for a broader range of macromolecular therapeutics.

## 7 Conclusions

Reverse micelles provide a versatile strategy to coat (poly) peptide (pP) drugs with a lipophilic layer, enabling their incorporation into lipid‐based nanocarriers. In contrast to hydrophobic ion pairing, this approach does not require the formation of defined ionic complexes and can instead exploit the organized surfactant interface, where electrostatic and other non‐covalent interactions contribute to pP stabilization, offering significantly greater flexibility. Moreover, the use of cationic surfactants—often associated with safety concerns—can be avoided. As a result of this lipophilic coating, active pharmaceutical ingredients are protected from enzymatic degradation by gastrointestinal peptidases and proteases, while their membrane permeability is markedly enhanced. Together, these benefits provide distinct formulation flexibility and support favorable oral bioavailability profiles for pP therapeutics, highlighting reverse micelles as a promising platform for the future development of non‐invasive delivery systems.

## Author Contributions

**Marlene Ramona Schmidt**: Writing – review & editing, Writing – original draft, Conceptualization. **Melanie Lena Ebert**: Writing – review & editing, Writing – original draft, Conceptualization. **Andreas Bernkop‐Schnürch**: Supervision, Conceptualization, Project administration, Funding acquisition, Writing – review & editing.

## Declaration of Generative AI and AI‐Assisted technologies in the Writing Process

During the preparation of this work, the authors used ChatGPT (OpenAI), Perplexity AI & Microsoft Copilot in order to support language refinement and structural editing of selected sections of the text. After using this tool/service, the authors reviewed and edited the content as needed and take full responsibility for the content of the publication.

## Conflicts of Interest

The authors declare no conflicts of interest.

## Data Availability

The data that support the findings of this study are available from the corresponding author upon reasonable request.
